# Development and validation of potential molecular subtypes and signatures of ocular sarcoidosis based on autophagy-related gene analysis

**DOI:** 10.1515/med-2025-1243

**Published:** 2025-08-05

**Authors:** Zixuan Wu, Xi Long, Kang Tan, Xiaolei Yao, Qinghua Peng

**Affiliations:** Hunan University of Traditional Chinese Medicine, Changsha, Hunan, 410208, China; Department of Ophthalmology, The First Affiliated Hospital of Hunan University of Traditional Chinese Medicine, Changsha, Hunan, 410007, China

**Keywords:** ocular sarcoidosis, autophagy-related genes, biomarker candidates, immune infiltration, cell communication

## Abstract

**Background:**

Sarcoidosis is characterized by the proliferation of noncaseating granulomas and presents as a complex chronic inflammatory disease. Autophagy plays a crucial role in the initiation, progression, and treatment resistance of various cancers. Despite the recognized importance of autophagy, the involvement of autophagy-related genes (ARGs) in the pathophysiology of ocular sarcoidosis (OS) remains largely unexplored.

**Methods:**

We intersected differentially expressed genes with a curated list of 177 ARGs to identify candidates potentially involved in OS. Advanced methodologies, including GSEA and GSVA, were employed to explore the biological functions. Further refinement using Lasso regression and SVM-RFE allowed for the identification of key hub genes and the assessment of their diagnostic potential for OS.

**Results:**

Our investigation identified 11 ARGs (DRAM1, SOGA1, ATG16L2, FYCO1, ATG7, ATG12, ATG14, KIAA0226, KIAA1324, KIAA1324L, and KIAA0226L) closely associated with OS. Functional analyses revealed their involvement in processes such as extracellular stimulus, response to nutrient levels, and positive regulation of catabolic process. Importantly, the diagnostic capabilities of these ARGs demonstrated significant efficacy in distinguishing OS from unaffected states.

**Conclusions:**

Through rigorous bioinformatics analyses, this study identifies 11 ARGs as novel biomarker candidates for OS, elucidating their potential roles in the disease’s pathogenesis.

## 1 Introduction

Sarcoidosis is a systemic granulomatous disorder marked by non-caseating epithelioid granulomas across multiple organs. Its etiology remains poorly understood, arising from complex interactions between immune dysregulation and environmental exposures, particularly infectious agents [1]. Ocular sarcoidosis (OS) reflects the clinical heterogeneity of the disease, ranging from isolated ocular involvement to manifestations that precede or accompany systemic disease [2]. Diagnosis remains challenging due to the variable sensitivity of current tests and the frequent reliance on tissue biopsy for definitive histological confirmation [3]. Ocular involvement occurs in approximately 7–60% of cases, with posterior segment disease often portending central nervous system involvement [4]. The diagnostic process is further complicated by the clinical mimicry of OS with other ocular inflammatory conditions [5]. Histopathologically, the identification of non-necrotizing granulomas and the exclusion of infectious or foreign material by negative staining remain the gold standard. Given these diagnostic limitations, elucidating the molecular underpinnings of OS is critical [6]. A mechanistic understanding of its pathophysiology is key to developing targeted therapies that may reduce disease recurrence and improve clinical outcomes, ultimately advancing the management of sarcoidosis.

Autophagy is a conserved cellular process that degrades cytoplasmic components via the lysosomal pathway, playing a pivotal role in cellular self-renewal, homeostasis, and – in certain contexts – programmed cell death [7,8]. Mounting evidence highlights its essential function in preserving intracellular integrity and regulating diverse physiological processes. Dysregulation of autophagy, whether through excessive activation or suppression, has been implicated in the pathogenesis of numerous diseases, including cancer and pulmonary disorders. While autophagy-related genes (ARGs) are known to influence tumor initiation and progression, their precise contributions remain incompletely characterized [9,10]. Beyond its metabolic and catabolic roles, autophagy is intricately involved in immune regulation. It is essential for antigen processing and presentation via major histocompatibility complex class II molecules, thereby facilitating T-cell activation and adaptive immune responses [11]. In the innate immune system, autophagy modulates the activity of macrophages and dendritic cells, enhancing their capacity to detect and eliminate pathogens. Notably, autophagy intersects with apoptotic pathways, shaping immune cell survival and function [12]. During lymphocyte development, it contributes to the clearance of aberrant or superfluous cells, ensuring immune homeostasis. Conversely, impaired autophagy is associated with autoimmune and infectious diseases, where pathogens may evade immune clearance by subverting autophagic mechanisms [13]. Deciphering the intricate crosstalk between autophagy and immunity is crucial for advancing novel immunotherapeutic strategies.

**Figure 1:**
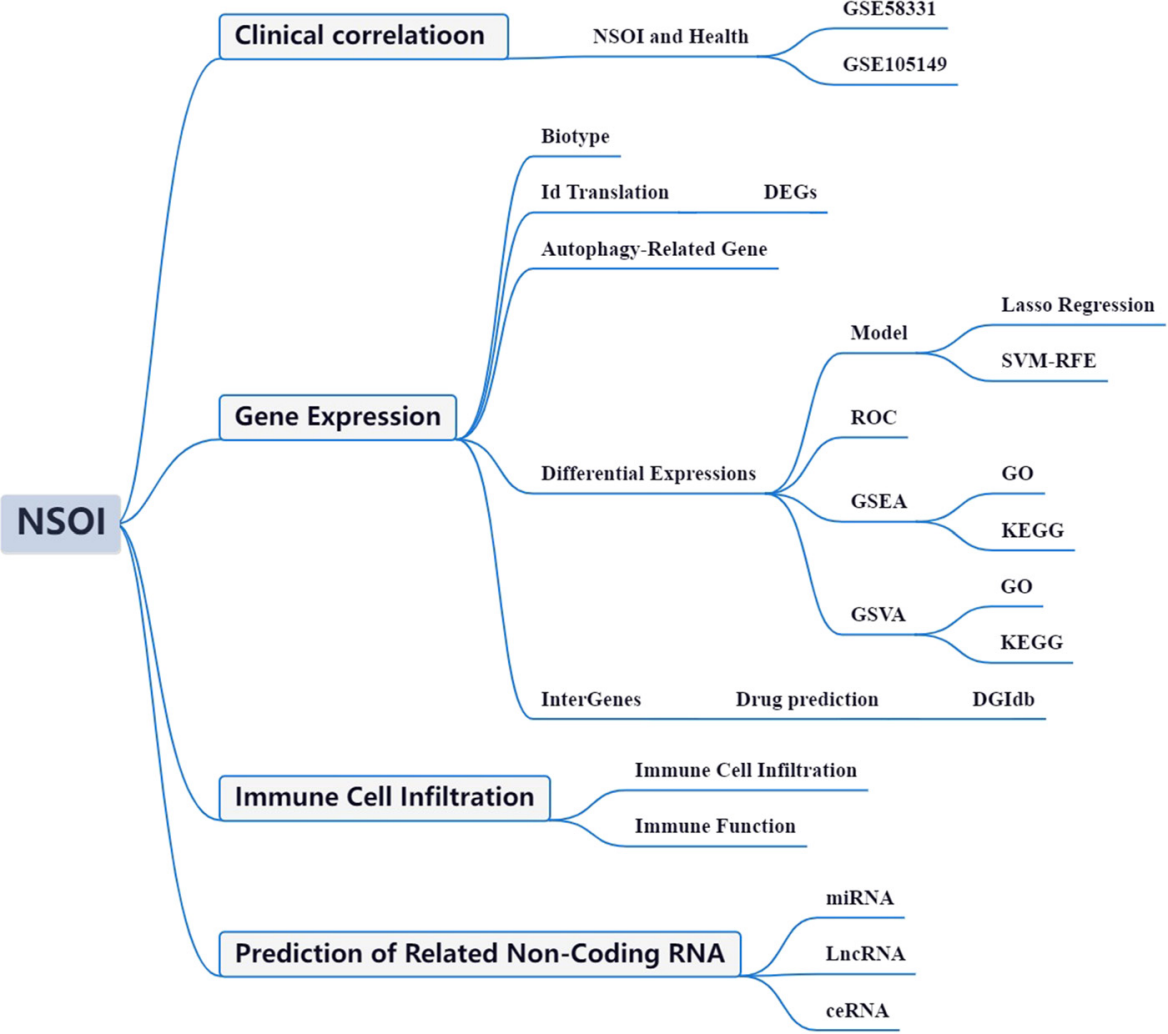
Framework.

The advent of high-throughput transcriptomic sequencing, combined with comprehensive clinical annotations from the OS Initiative, has enabled unprecedented exploration of the transcriptional dynamics and molecular networks underlying OS [14,15,16]. Bioinformatic interrogation of these rich datasets has yielded critical insights into the multifactorial pathophysiology of OS. However, the specific roles of ARGs in OS remain underexplored. To address this gap, the present study aimed to systematically analyze OS-associated GEO datasets to elucidate the functional relevance of ARGs in OS pathogenesis, as illustrated in Figure 1.

## 2 Materials and methods

We adopted the methodologies delineated by Wu et al. in 2023 [17].

### 2.1 Raw data and differentially expressed genes (DEGs)

We utilized two foundational datasets from the GEO series, GSE58331 and GSE105149. GSE58331 was used for training, while GSE105149 was reserved for validation. Additionally, the MSigDB provided a comprehensive list of 307 ARGs (Table S1). mRNA profiles were extracted using Perl scripts to match and sort transcriptional data from GSE58331. Following normalization, DEGs among the ARGs were identified using stringent criteria: FDR < 0.05 and |log2FC| ≥ 1. Pearson’s correlation coefficient was then employed, using the corrplot package in R.

### 2.2 Development of a predictive model, immune cell infiltration, and functional enrichment analysis

To explore the biological relevance and pathway engagement of DEGs, we performed GO and KEGG analyses. We utilized the R programming environment to investigate the influence of differentially expressed ARGs on BP, MF, and CC. For model optimization, Lasso regression analysis was implemented using the glmnet package, enhanced through rigorous cross-validation procedures. Further validation was achieved through the deployment of the SVM-RFE algorithm, facilitated by the e1071 package, meticulously crafting a robust machine learning model. The reliability and accuracy of our model were stringently assessed via cross-validation, focusing on minimizing error rates and ensuring predictive consistency. Through the utilization of the ggplot2 package, our analysis prioritized DEGs, pinpointing pivotal genes crucial for disease classification. Moreover, the employment of the CIBERSORT algorithm enabled detailed analysis of immune cell composition, providing profound insights into the immunological milieu pertinent to the pathology under investigation.

### 2.3 Gene set enrichment and variation analyses and drug–gene interaction insights

To decipher functional dynamics and pathway deviations in diverse biological samples, we applied GSEA and GSVA. Using the R platform, we analyzed the impact of differentially expressed ARGs on BP, MF, and CC and related pathways. This provided a comprehensive view of their involvement in disease pathophysiology. Recognizing the critical role of validated biomarkers in therapeutic design, precise drug–gene interaction predictions are paramount. For this purpose, we leveraged the DGIdb to forecast potential interactions for pivotal hub genes, thereby informing potential therapeutic targets.

### 2.4 Assembly of an mRNA–miRNA–lncRNA regulatory network

Non-coding RNA transcripts are crucial determinants in the genetic regulatory framework. miRNAs orchestrate gene expression by modulating mRNA stability and translation, whereas lncRNAs, which exceed 200 nucleotides in length, influence cellular mechanisms through chromosomal modifications, transcriptional activation, and molecular interference. Recent evidence underscores a sophisticated interaction between miRNAs and lncRNAs, manifesting in competitive binding dynamics with other regulatory molecules. This interaction model, referred to as the ceRNA hypothesis, elucidates how lncRNAs modulate gene expression by miRNA sequestration. To construct this regulatory network, we extracted validated miRNA–lncRNA–target interactions from databases such as miRTarBase and PrognoScan, providing a rich resource for understanding these complex regulatory relationships.


**Ethical approval:** This manuscript is not a clinical trial; hence, the ethics approval and consent to participation are not applicable.

## 3 Results

### 3.1 Identification and enrichment analysis of DEGs

**Figure 2:**
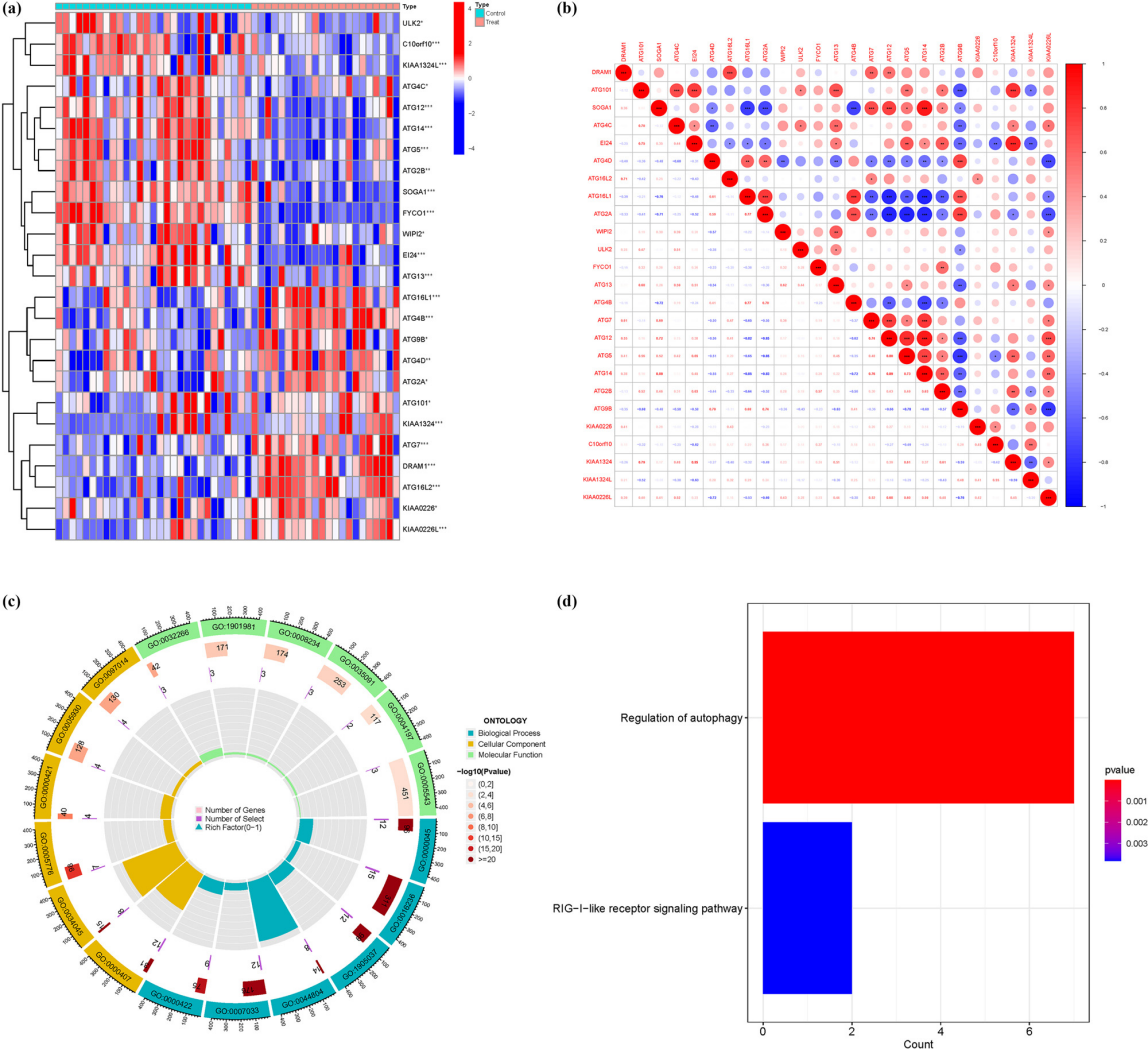
Principal component analysis. (a) Difference analysis. (b) Correlation analysis. (c) GO. (d) KEGG.

**Figure 3:**
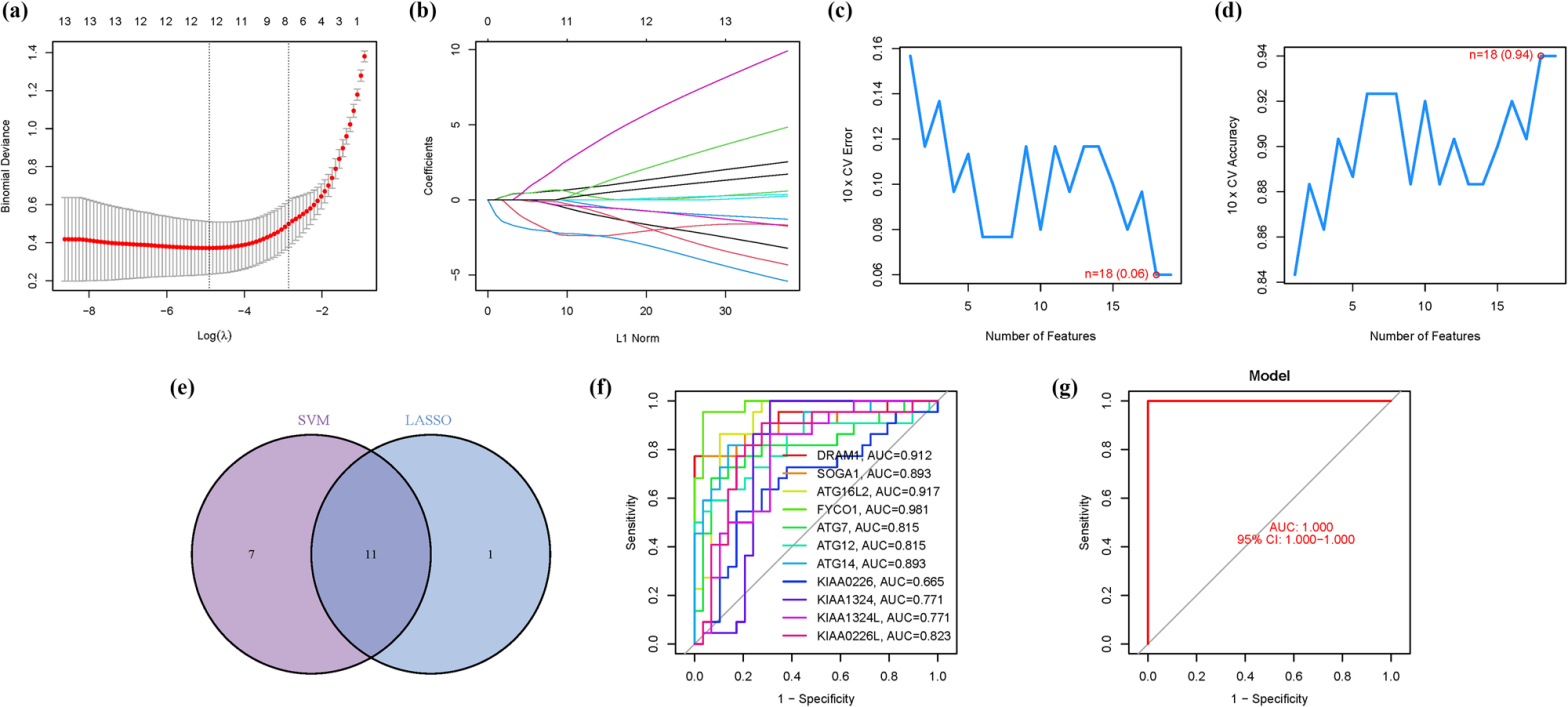
The ARG signature. (a) Regression of Lasso. (b) Cross-validation. (c and d) Accuracy and error. (e) Venn. (f) AUC of 11 hub genes. (g) AUC of the training group.

Among the 25 ARGs analyzed, a subset showed significant differences in expression levels between treatment and control groups, specifically genes such as ATG2A, ATG101, KIAA1324, ATG7, DRAM1, ATG16L2, KIAA0226, and KIAA0226L in the treatment group, while genes like TG4C, ATG12, ATG14, ATG5, ATG2B, SOGA1, FYCO1, and WIPI2 in the control group (Figure 2a). These DEGs were further analyzed for correlation, and a correlation matrix was visualized (Figure 2b) (Table S2). GO enrichment analysis identified 120 core targets. MFs primarily included phospholipid binding (GO:0005543), phosphatidylinositol binding (GO:0035091), and GTPase binding (GO:0051020). CCs are mainly involved in vacuolar membrane (GO:0005774), endocytic vesicle (GO:0030139), and extrinsic component of membrane (GO:0019898). BPs mainly included response to extracellular stimulus (GO:0009991), response to nutrient levels (GO:0031667), and positive regulation of catabolic process (GO:0009896). KEGG analysis revealed that overexpressed genes were primarily involved in the regulation of autophagy (hsa04140) and RIG-I-like receptor signaling pathway (hsa04622) (Figure 3 and Tables S3 and S4).

### 3.2 Model construction

We employed a comprehensive methodology involving Lasso regression analysis and Cox proportional hazards regression analysis (Figure 3a and b). Subsequently, we constructed a machine learning model using SVM-RFE to validate the predictive accuracy and reliability of the model. This model demonstrated impressive accuracy (0.94) with a minimal error rate of 0.06 (Figure 3c and d). A cross-reference of five ARGs identified through both Lasso and SVM methodologies was performed (Figure 3e). Comparative analysis with these five hub genes revealed significantly high area under the curve (AUC) values, indicating robust predictive power: DRAM1 (AUC = 0.912), SOGA1 (AUC = 0.893), ATG16L2 (AUC = 0.917), FYCO1 (AUC = 0.981), ATG7 (AUC = 0.815), ATG12 (AUC = 0.815), ATG14 (AUC = 0.893), KIAA0226 (AUC = 0.665), KIAA1324 (AUC = 0.771), KIAA1324L (AUC = 0.771), and KIAA0226L (AUC = 0.823) (Figure 3f). Notably, an AUC of 1.000 (95% confidence interval [CI]: 1.000–1.000) was achieved in the GSE58331 dataset (Figure 3g; Table 1 and Table S4).

**Table 1:** The characteristics of model

Label	Lasso	SVM-RFE
Sensitivity	1	1
Specificity	0.875	0.75
Pos pred value	0.916666667	0.846153846
Neg pred value	1	1
Precision	0.916666667	0.846153846
Recall	1	1
*F*1	0.956521739	0.916666667
Prevalence	0.578947368	0.578947368
Detection rate	0.578947368	0.578947368
Detection prevalence	0.631578947	0.684210526
Balanced accuracy	0.9375	0.875

### 3.3 GSEA analysis

**Figure 4:**
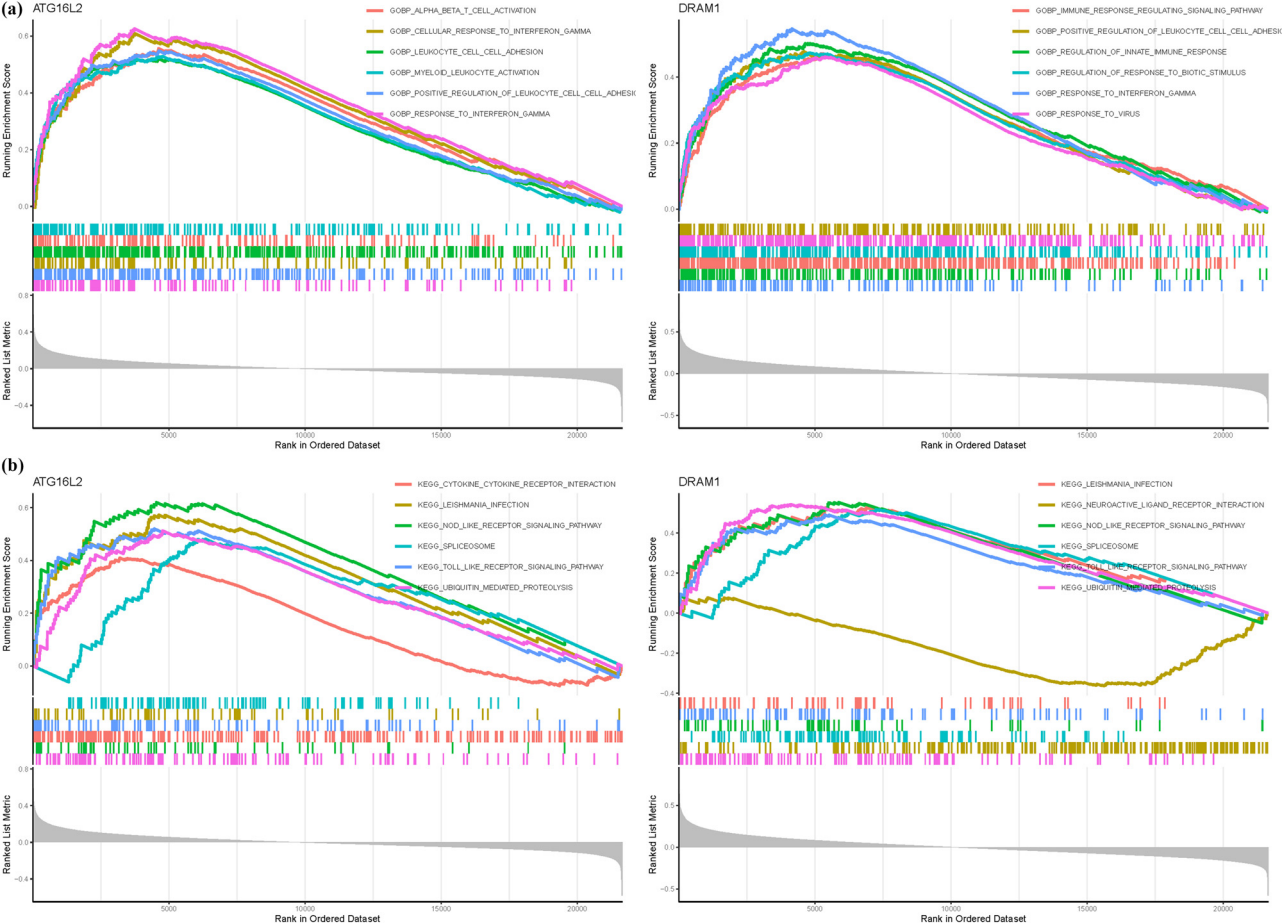
GSEA analysis in ATG16L2 and DRAM1. (a) GO. (b) KEGG.

Through literature review and sensitivity analysis within the model, we identified ATG16L2 and DRAM1 as potentially the most relevant genes to OS. In terms of GO analysis, ATG16L2 mainly involves BP cellular response to interferon gamma, BP leukocyte cell–cell adhesion, and BP myeloid leukocyte activation. DRAM1 mainly involves BP regulation of innate immune response, BP regulation of response to biotic stimulus, and BP response to interferon gamma (Figure 4a). In KEGG analysis, ATG16L2 is primarily associated with leishmania infection, nod-like receptor signaling pathway, and spliceosome, while DRAM1 is linked to nod-like receptor signaling pathway, spliceosome, and toll-like receptor signaling pathway (Figure 4b) (Table S5).

### 3.4 Immunological environment in OS

**Figure 5:**
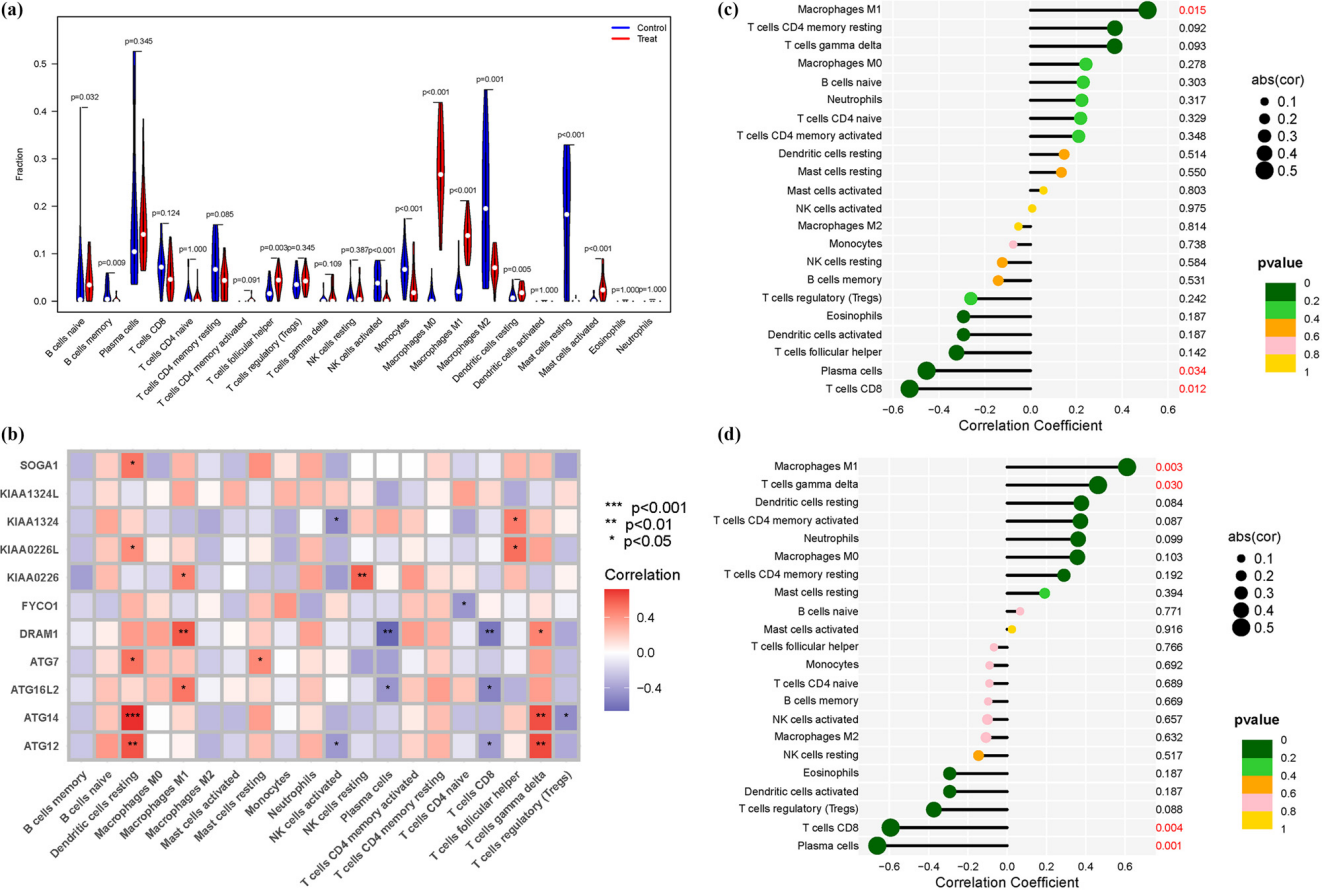
Immunological environment. (a) Immune cell. (b) Correlation between ARGs and immune cells. (c) ATG16L2. (d) DRAM1.

The immunological milieu plays a pivotal role in the initiation and progression of OS. We utilized a violin plot to depict the distribution of immune cell levels. In the treatment group, there was a marked elevation in the expression of naive B cells, memory B cells, follicular helper T cells, resting NK cells, M0 macrophages, and activated mast cells. Conversely, the control group showed significantly higher levels of memory cells, activated NK cells, M2 macrophages, and resting mast cells (Figure 5a). Further, we performed a correlation analysis between these genes and immune cells (Figure 5b). Additionally, we separately analyzed the immune infiltration patterns of ATG16L2 and DRAM1 (Figure 5c and d).

### 3.5 GSVA analysis

**Figure 6:**
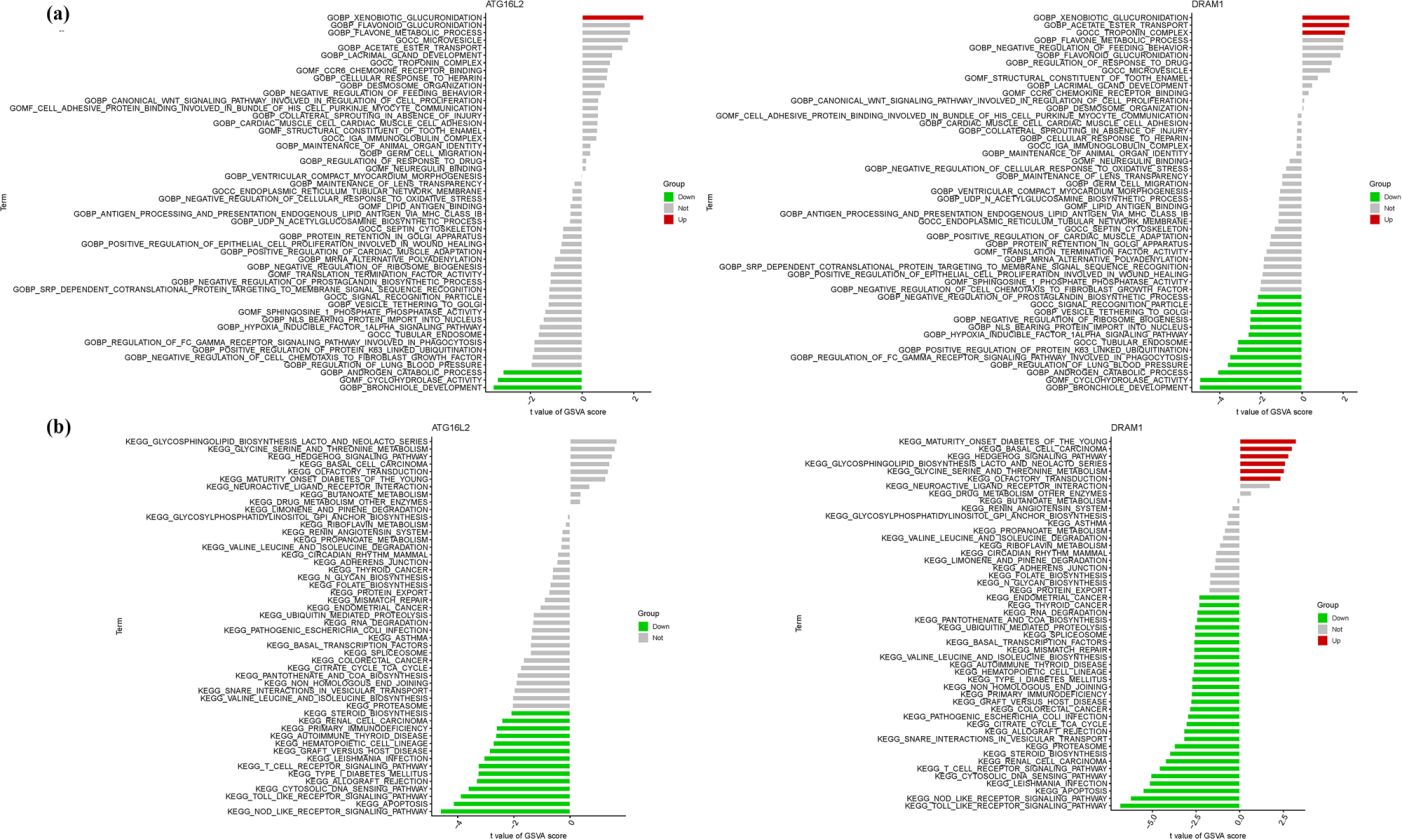
GSVA analysis in ATG16L2 and DRAM1. (a) GO. (b) KEGG.

ATG16L2 mainly involves BP cellular response to heparin, MF CCR6 chemokine receptor binding, CC troponin complex, BP lacrimal gland development, BP acetate ester transport, CC microvesicle, BP flavone metabolic process, BP flavonoid glucuronidation, and BP xenobiotic glucuronidation. DRAM1 mainly involves MF structural constituent of tooth enamel, CC microvesicle, BP regulation of response to drug, BP flavonoid glucuronidation, BP negative regulation of feeding behavior, BP flavone metabolic process, CC troponin complex, BP acetate ester transport, and BP xenobiotic glucuronidation (Figure 6a). In terms of KEGG analysis, ATG16L2 mainly involves limonene and pinene degradation, drug metabolism – other enzymes, butanoate metabolism, neuroactive ligand receptor interaction, maturity onset diabetes of the young, olfactory transduction, basal cell carcinoma, hedgehog signaling pathway, glycine serine and threonine metabolism, and glycosphingolipid biosynthesis lacto and neolacto series. DRAM1 mainly involves butanoate metabolism, drug metabolism – other enzymes, neuroactive ligand receptor interaction, olfactory transduction, glycine serine and threonine metabolism, glycosphingolipid biosynthesis lacto and neolacto series, hedgehog signaling pathway, basal cell carcinoma, maturity onset diabetes of the young (Figure 6b).

### 3.6 Drug–gene interactions and construction of miRNA–lncRNA shared genes network

**Figure 7:**
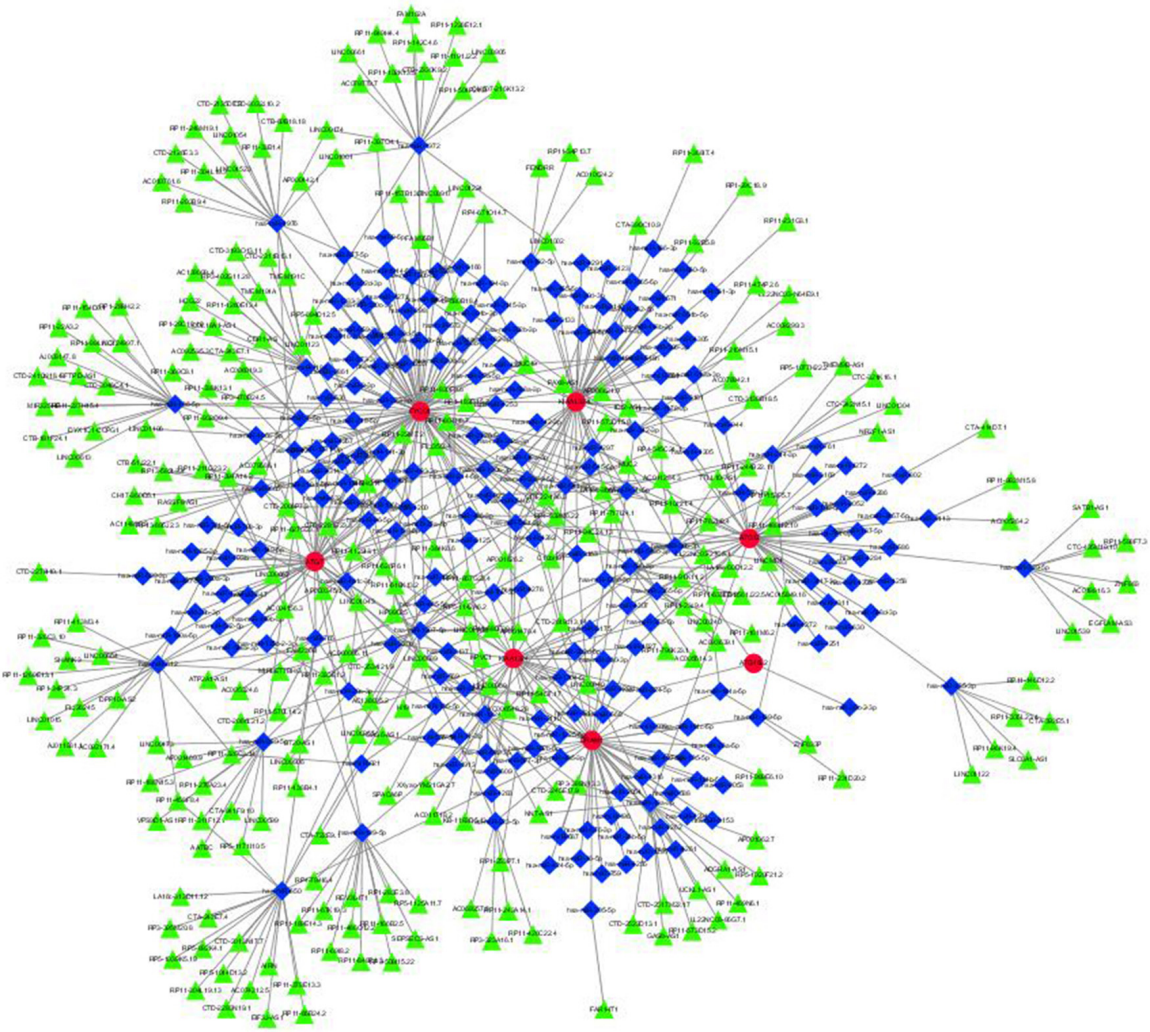
miRNAs–lncRNAs shared genes network. Note: Red circles are mRNAs, blue quadrangles are miRNAs, and green triangles are lncRNAs.

The 11 hub genes predicted one drug (cisplatin). We conducted searches in three databases to identify 289 miRNAs and 376 lncRNAs linked with OS (Tables S10 and S11). Table S6 demonstrates the matching of these genes against the corresponding miRNA database. The databases used for this search were miRanda [18], miRDB [19], and TargetScan [20]. Each match with the relevant miRNA in these databases was assigned a score of 1. Notably, a match in all three databases received a score of 3. The corresponding lncRNA data were obtained by matching the miRNAs with the spongeScan database. The miRNA–lncRNA–gene interaction network was established by intersecting datasets with shared genes identified through Lasso regression and SVM-RFE. This comprehensive network encompasses 271 lncRNAs, 225 miRNAs, and several common genes, prominently featuring 7 hub genes: FYCO1, KIAA1324L, ATG7, KIAA1324, DRAM1, ATG16L2, and ATG12 (Figure 7).

### 3.7 Validation of hub genes

**Figure 8:**
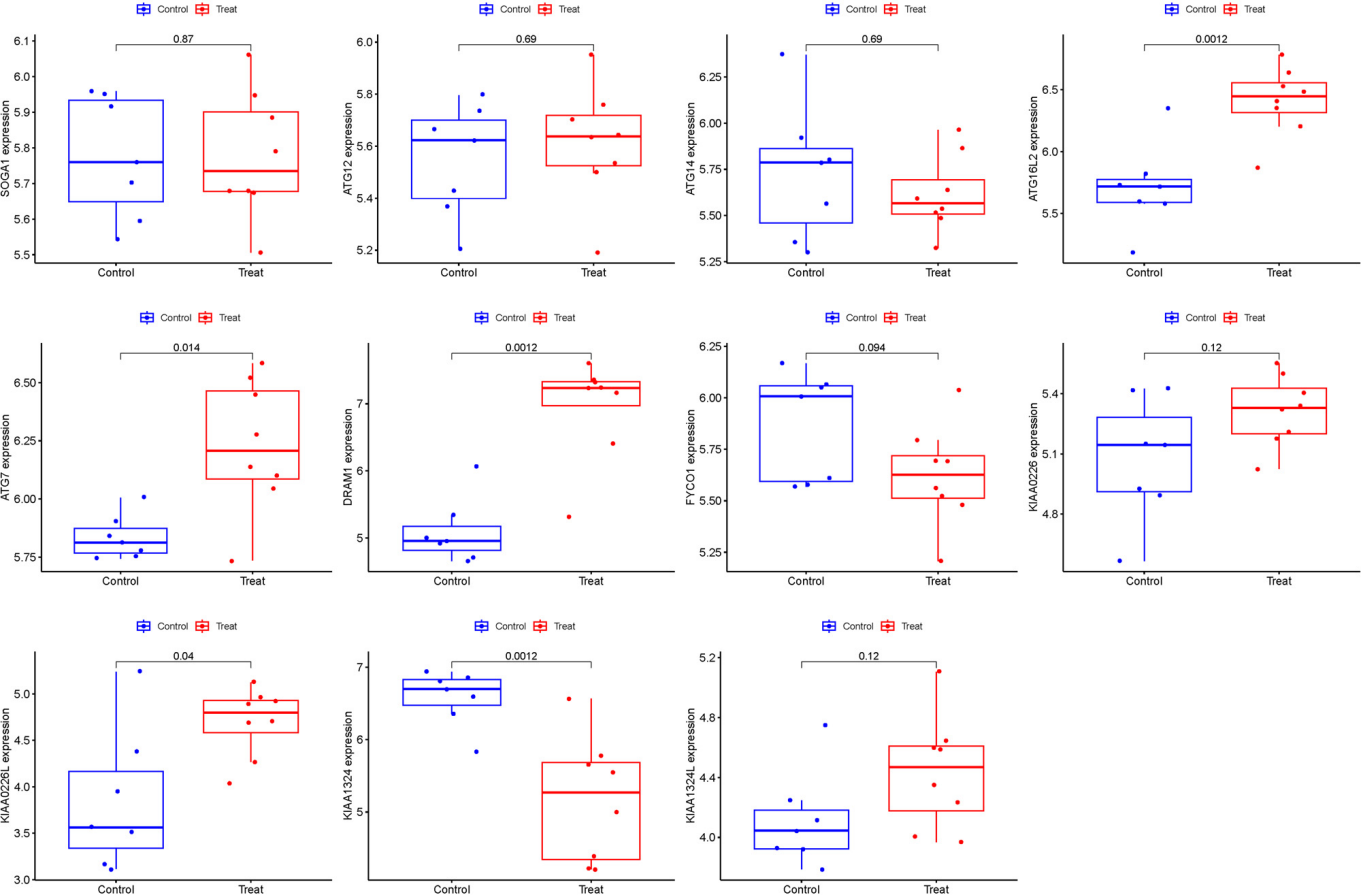
Validation of hub genes.

We used GSE105149 for validation of the hub genes. However, among the five ARGs, only ATG16L2 and DRAM1 showed significant differences in the GSE105149 analysis (Figure 8). Upon recalibration of the data, we observed differences in sample sizes between the two datasets, as well as differences in patient sources, which may have contributed to bias in the results.

### 3.8 Model verification

**Figure 9:**
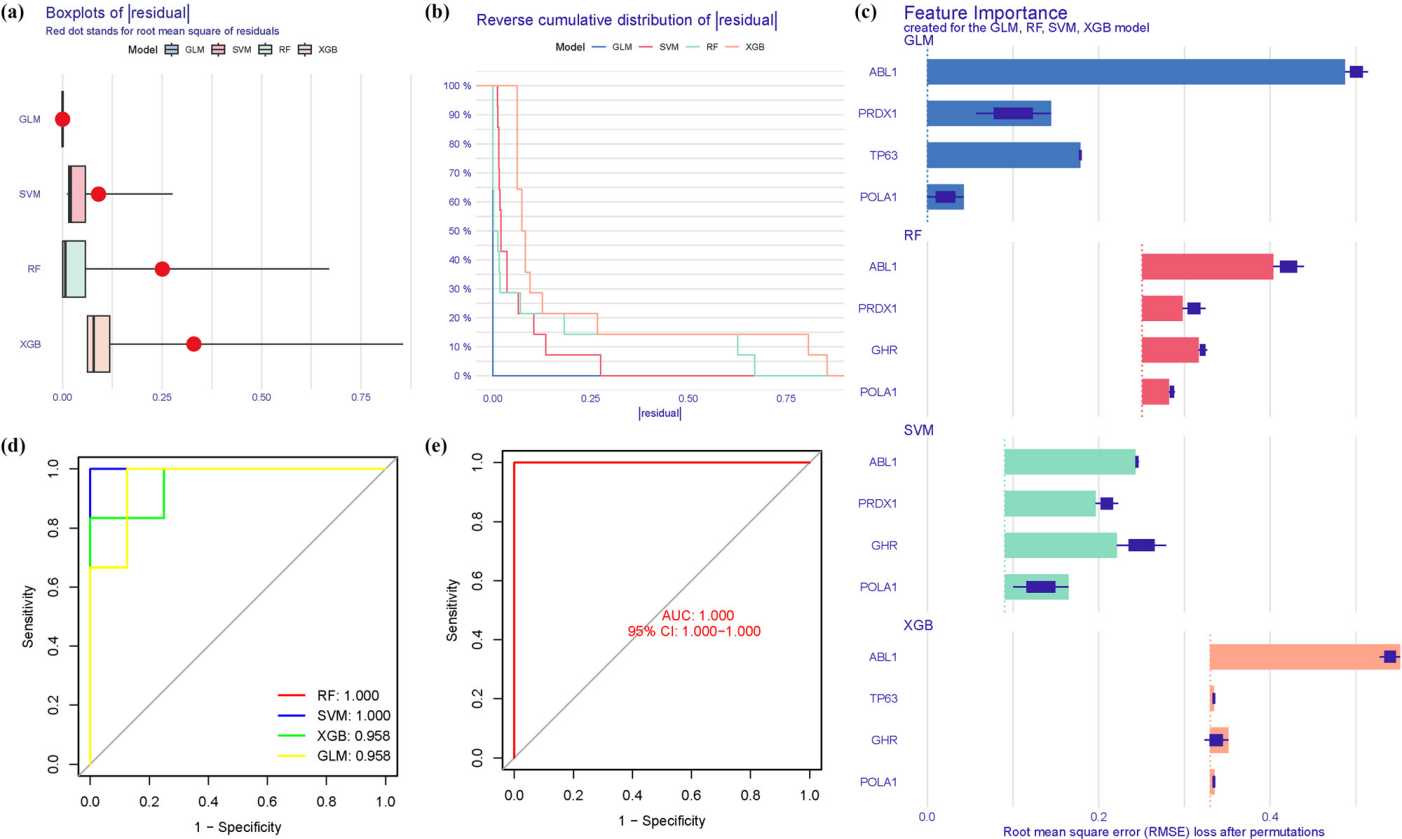
Model verification. (a) Residual expression patterns. (b and c) Model expression patterns. (d) AUC of the model. (e) AUC of the test group.

The boxplots illustrated the residual expression patterns of these genes in OS (Figure 9a). There were differences in the proportions of the four different modes (Figure 9b and c). The diagnostic capacity of the ARGs in distinguishing OS from control samples revealed a satisfactory diagnostic value, with an AUC of RF: 1.000; SVM: 1.000; XGB: 1.000; and GLM: 1.000 (Figure 9d). Additionally, an AUC of 1.000 (95% CI 1.000–1.000) was achieved in GSE105149 (Figure 9e).

## 4 Discussion

Ophthalmic disturbances are prevalent in a significant proportion of sarcoidosis patients, affecting 30–60% of cases. OS may occur independently, without systemic manifestations, or as the primary site of affliction, accompanied by limited symptoms in other regions [21]. Although OS can affect any part of the eye, uveitis is the most common form, impacting 20–30% of those diagnosed with sarcoidosis [22]. The burden of these symptoms is considerable, affecting patients’ physical and psychological well-being and imposing substantial economic costs. Ocular complications can lead to reduced visual function, pain, discomfort, and diminished quality of life [23]. Addressing the challenges of OS necessitates a multidisciplinary approach involving ophthalmologists, pulmonologists, rheumatologists, and other relevant specialists to ensure comprehensive care. Accurate and timely diagnosis of OS is critical to prevent irreversible damage and optimize treatment outcomes [24]. Autophagy, as a critical intracellular degradation and recycling mechanism, maintains the health of ocular tissues by regulating cellular homeostasis and removing damaged organelles and proteins. In glaucoma, autophagy mitigates neuronal death and optic nerve damage by clearing damaged retinal ganglion cells and mitochondria. Studies have shown that activation of autophagy helps protect retinal cells, reducing cellular stress and damage caused by elevated intraocular pressure [25]. In age-related macular degeneration (AMD), impaired autophagy is considered a key factor in the degeneration and death of retinal pigment epithelial cells. Defective autophagy may lead to the accumulation of harmful intracellular substances, exacerbating retinal damage and macular degeneration [26]. Enhancing autophagic activity is viewed as a potential therapeutic strategy to clear accumulated pathogenic proteins and lipids, thereby protecting retinal cells. Moreover, in diabetic retinopathy, autophagy plays a crucial role. Under hyperglycemic conditions, dysregulated autophagy may contribute to retinal vascular abnormalities and neurodegenerative changes. Modulating autophagy pathways could effectively alleviate retinal damage caused by diabetes and protect visual function [27]. In summary, autophagy is pivotal in the pathogenesis and progression of various ocular diseases. A deeper understanding of the relationship between autophagy and eye diseases will aid in the development of novel therapeutic approaches to preserve vision and ocular health. Our investigation elucidated the significant interactions between these genes and specific transcription factors within the context of angiogenesis. A thorough review of existing literature highlighted the pivotal roles of ATG16L2 and DRAM1 genes in the interface between OS and ARGs. Further analysis of their biological functions demonstrated their involvement in various immune-related processes, such as epithelial cell migration, tissue migration, and broader epithelium dynamics. These findings suggest that ARGs may exert extensive regulatory influence across diverse biological pathways, particularly in immune modulation.

ATG16L2 and DRAM1 are genes that have been increasingly recognized for their roles in a variety of pathological conditions, including ocular diseases. Their involvement in extracellular matrix remodeling and immune responses, respectively, underscores their importance in disease mechanisms. The genes ATG16L2 and DRAM1 have been identified as significant contributors to the pathogenesis of various diseases through their roles in autophagy. The ATG16L2 gene encodes a protein that is integral to the autophagy process, which is essential for maintaining cellular homeostasis and removing damaged organelles [28]. Dysregulation of ATG16L2, due to mutations or altered expression, can lead to autophagic dysfunction, which is implicated in a range of disorders, including AMD and retinitis pigmentosa [29]. DRAM1, another key player in autophagy, encodes a protein that is critical for the formation and maturation of autophagosomes. Functional impairments in DRAM1 are associated with the pathogenesis of several degenerative diseases, notably glaucoma and diabetic retinopathy. In these conditions, abnormal DRAM1 expression can induce cellular stress and apoptosis, thereby exacerbating disease progression [30]. Thus, aberrations in ATG16L2 and DRAM1 may facilitate the development of diseases by disrupting autophagic pathways. These discoveries not only deepen our understanding of the molecular mechanisms driving these conditions but also suggest potential therapeutic targets. Continued research is essential to elucidate the precise roles of these genes across various pathological states and to assess their viability as therapeutic targets. This body of evidence reveals a nuanced interplay between ATG16L2 and DRAM1 in regulating retinal function and intraocular pressure, offering fresh insights into the pathophysiology of ocular diseases. Our study, supported by dataset GSE105149, delves into the roles of these genes in OS, suggesting that angiogenesis-related traits could serve as potential prognostic indicators. This comprehension paves the way for novel approaches in managing and treating ocular diseases, underscoring the importance of ATG16L2 and DRAM1 in preserving ocular health.

In the intricate landscape of OS, an expanding body of evidence is challenging the traditional view that links an enhanced immune response solely to CD4 cell activity. Instead, a more complex picture is emerging, one that includes pre-existing T-regulatory cells and a blend of both proinflammatory and regulatory elements, such as variations in cytokine levels [31]. This complexity may underlie a disordered immune reconstitution, rendering individuals more vulnerable to opportunistic infections – whether existing, latent, or previously managed [32]. As a result, diseases such as tuberculosis, cytomegalovirus infections, progressive multifocal leukoencephalopathy, kaposis sarcoma, and various autoimmune disorders may become exacerbated or escape detection. Notably, cytomegalovirus retinitis emerges as a leading opportunistic infection associated with immunological recovery uveitis [33,34]. Emerging therapeutic strategies that aim to increase intracellular cAMP levels offer a promising avenue for mitigating chronic inflammation. Small-molecule phosphodiesterase 4 inhibitors, which prevent cAMP degradation, have shown efficacy in a range of inflammatory conditions, including inflammatory bowel disease, atopic dermatitis, and rheumatoid arthritis [35,36]. Expanding on our prior research, we investigated the expression patterns of ARGs in the immune environment. Strikingly, our observations revealed significant expression in various cell types within the treated group, including naive B cells, memory B cells, follicular helper T cells, resting NK cells, etc. Through bioinformatics validation, we identified abnormal gene expression signatures linked to dysregulated immune responses in OS patients, offering crucial insights into the disease mechanisms.

The exploration of biomarkers in the context of OS has been notably limited, with the intricate relationship between metabolic processes and ocular diseases only recently beginning to emerge through bioinformatics analyses [37,38,39]. In the rapidly evolving field of OS research, Liu et al. recently identified hub genes associated with the condition using WGCNA. Similarly, Hu et al. developed a bioinformatics model for thyroid eye disease, pinpointing 11 key genes. Huang et al. made significant strides by identifying six crucial genes central to diabetic retinopathy through sophisticated bioinformatics analyses, complemented by *in vivo* validation. Despite these advancements, the intersection between angiogenesis and OS remains largely unexplored. Our study aims to address this gap by investigating cell metabolism and examining ARGs extracted from GEO datasets. This innovative approach distinguishes our work, offering novel theoretical perspectives and methodological innovations. However, our study has limitations. A deeper understanding of the molecular dynamics linking ARGs with OS is crucial. Both *in vivo* and *in vitro* studies hold immense potential for unraveling these complexities, suggesting numerous avenues for future research. Additionally, exploring the correlation between prognostic genes and ARGs in the context of OS is essential.

## 5 Conclusion

The pathogenesis and progression of OS involve complex, multifactorial interactions among various targets, pathways, signaling molecules, and regulatory frameworks. Among these, ATG16L2 and DRAM1 are pivotal, exerting significant influence on the angiogenesis program.

## Supplementary Material

Supplementary Table
