# Supplementary material for: Development and validation of potential molecular subtypes and signatures of ocular sarcoidosis based on autophagy-related gene analysis
Source: Open Med (Wars). 2025 Aug 5;20(1):20251243. doi: 10.1515/med-2025-1243 (PMC12326308; doi:10.1515/med-2025-1243)
Supplement: Supplementary Table [file med-2025-1243-sm.pdf]

Supplementary material

Appendix 1

A1 Datasets and autophagy-related Gene

Table S1: Autophagy-related gene

DRAM1	AMBRA1	APG4A	KIAA0889	MGD3208
ATG101	ATG4B	ATG16B	PRO180	APG12L
SOGA1	ATG7	APG16L	ATG18A	APG5-LIKE
DRAM2	ATG9A	1A1-3B	KIAA1324	BARKOR
WIPI1	ATG12	ATG18B	KIAA1324L	APG10L
ELAPOR1	ATG5	KIAA0226	KIAA0226L	KIAA1632
ELAPOR2	ATG14	APG3	APG4C	ATG1A
RUBCNL	ATG2B	C10orf10	PIG8	NOS3AS
ATG4C	ATG10	ATG1B	APG4D	SOGA
EI24	EPG5	CATC2	AUTL2	TMEM77
ATG4D	ULK1	KIAA0652	WDR80	WIPI49
ATG4A	ATG9B	DCAF3	ATG16A	PACER
ATG16L2	DRAM	APG4B	IAI3B	TP53I8
ATG16L1	C12orf44	APG7-LIKE	Atg21	AUTL4
ATG2A	C20orf117	APG9L1	RUBICON	WWFQ154
NBR1	CORD21	APG12	APG3-LIKE	AUTL3
WIPI2	ATG18	APG5	DEPP	ATG16L
RUBCN	EIG121	ATG14L	Unc51.2	IBD10
ATG3	EIG121L	C14orf103	CTRCT18	M17S2
DEPP1	C13orf18	APG10	PARATARG8	CGI-50
ULK2	APG4-C	HEEW1	WDR94	SCAR15
FYCO1	EPG4	ATG1	AUTL1	APG3L
ATG13	APG4-D	APG9L2	APG7L	FIG
ASP	IDDSSA	KIAA0831	Fseg	WDR30
SCAR25	ZFYVE7	pp12616	GSA7	RUFY3
hAPG5	SCAR31	VICIS	mATG9	WIPI-2
hATG1	HAPG12	UNC51	FBR93	PC3-96
Unc51.1	MIG19	SONE	APG5L	

Appendix 2

A2 DEGs linked to ARGs

Table S2: 25 DEGs linked to ARGs

Gene	conMean	treatMean	*p*value	Type
DRAM1	4.853020655	6.006647455	4.08×10^{-8}	Up
ATG101	5.796728552	6.021125	0.042813275	Up
SOGA1	5.803013793	5.362495636	1.90×10^{-6}	Down
ATG4C	3.380810241	3.053477045	0.046868236	Down
EI24	5.934531241	5.236245182	1.80×10^{-7}	Down
ATG4D	6.012636448	6.458845682	0.003829699	Up
ATG16L2	5.591473655	6.233995773	4.43×10^{-7}	Up
ATG16L1	3.644774897	4.169272591	0.000812745	Up
ATG2A	6.132250379	6.387083364	0.042091311	Up
WIPI2	5.64387069	5.517631409	0.02450768	Down
ULK2	3.671604759	3.445042727	0.015193534	Down
FYCO1	6.610282621	5.551617273	5.56×10^{-9}	Down
ATG13	6.584953966	6.250498318	0.000844323	Down
ATG4B	5.534146552	5.972706045	7.53×10^{-6}	Up
ATG7	5.231447172	5.604249864	7.02×10^{-5}	Up
ATG12	4.904496	4.241468273	7.02×10^{-5}	Down
ATG5	4.731144138	4.241763955	0.000596871	Down
ATG14	5.515735724	4.340343682	2.38×10^{-7}	Down
ATG2B	4.716425586	4.365553682	0.001541577	Down
ATG9B	3.544248241	3.779220273	0.044111147	Up
KIAA0226	4.918455448	5.020593182	0.046210557	Up
C10orf10	6.476110966	5.747226136	0.000618371	Down
KIAA1324	4.418637724	5.187288364	0.000752991	Up
KIAA1324L	3.867513103	3.4663905	0.000752991	Down

Appendix 3

A3 Analysis of enrichment

Table S3: Analysis of GO

Ontology	ID	Description	BgRatio	*p*value	*q*value
BP	GO:1905037	Autophagosome organization	99/18862	2.67×10^{-23}	1.89×10^{-21}
BP	GO:0051817	Modulation of process of other organism involved in symbiotic interaction	98/18862	0.000145406	0.001104208
BP	GO:0000045	Autophagosome assembly	96/18862	1.80×10^{-23}	1.89×10^{-21}
BP	GO:0042158	Lipoprotein biosynthetic process	94/18862	2.64×10^{-6}	3.50×10^{-5}
BP	GO:0006497	Protein lipidation	90/18862	2.21×10^{-6}	3.14×10^{-5}
BP	GO:0000422	Autophagy of mitochondrion	75/18862	2.45×10^{-17}	7.45×10^{-16}
BP	GO:0061726	Mitochondrion disassembly	75/18862	2.45×10^{-17}	7.45×10^{-16}
BP	GO:0061912	Selective autophagy	62/18862	3.68×10^{-11}	8.69×10^{-10}
BP	GO:0042149	Cellular response to glucose starvation	48/18862	0.049708566	0.117440121
BP	GO:0045058	T cell selection	48/18862	0.049708566	0.117440121
BP	GO:0009991	Response to extracellular stimulus	477/18862	0.000114666	0.000903019
BP	GO:0006111	Regulation of gluconeogenesis	47/18862	0.048697349	0.117440121
BP	GO:0090329	Regulation of DNA-dependent DNA replication	47/18862	0.048697349	0.117440121
BP	GO:0047496	Vesicle transport along microtubule	46/18862	0.047685109	0.117440121
BP	GO:0120009	Intermembrane lipid transfer	46/18862	0.047685109	0.117440121
BP	GO:0031667	Response to nutrient levels	451/18862	8.81×10^{-5}	0.000748961
BP	GO:0009896	Positive regulation of catabolic process	450/18862	0.011370863	0.057386208
BP	GO:0001974	Blood vessel remodeling	45/18862	0.046671847	0.117440121
BP	GO:0031057	Negative regulation of histone modification	45/18862	0.046671847	0.117440121
BP	GO:0010677	Negative regulation of cellular carbohydrate metabolic process	44/18862	0.04565756	0.117440121
BP	GO:1903214	Regulation of protein targeting to mitochondrion	44/18862	0.04565756	0.117440121
BP	GO:0097352	Autophagosome maturation	41/18862	0.000854423	0.005046593
BP	GO:0090207	Regulation of triglyceride metabolic process	39/18862	0.040570729	0.110597669
BP	GO:0044003	Modulation by symbiont of host process	37/18862	7.74×10^{-6}	9.14×10^{-5}
BP	GO:0090218	Positive regulation of lipid kinase activity	37/18862	0.038528794	0.106395301
BP	GO:0043687	Post-translational protein modification	361/18862	0.000501549	0.003136621
BP	GO:0042311	Vasodilation	36/18862	0.037506278	0.104934463
BP	GO:0039694	Viral RNA genome replication	35/18862	6.53×10^{-6}	8.17×10^{-5}
BP	GO:0043243	Positive regulation of protein-containing complex disassembly	35/18862	0.03648273	0.103431739
BP	GO:0019058	Viral life cycle	348/18862	0.005622602	0.031461651
BP	GO:0008156	Negative regulation of DNA replication	34/18862	0.035458147	0.101885431
BP	GO:0018215	Protein phosphopantetheinylation	331/18862	0.047384103	0.117440121
BP	GO:0032941	Secretion by tissue	33/18862	0.03443253	0.100293743
BP	GO:0043552	Positive regulation of phosphatidylinositol 3-kinase activity	33/18862	0.03443253	0.100293743

(*Continued*)

Table S3: *Continued*

Ontology	ID	Description	BgRatio	*p*value	*q*value
BP	GO:0010506	Regulation of autophagy	328/18862	8.34×10^{-7}	1.48×10^{-5}
BP	GO:0032984	Protein-containing complex disassembly	328/18862	0.04661544	0.117440121
BP	GO:0071312	Cellular response to alkaloid	32/18862	0.033405877	0.100044286
BP	GO:1903955	Positive regulation of protein targeting to mitochondrion	32/18862	0.033405877	0.100044286
BP	GO:0016236	Macroautophagy	311/18862	1.86×10^{-23}	1.89×10^{-21}
BP	GO:0071496	Cellular response to external stimulus	303/18862	0.000258136	0.001829598
BP	GO:0014072	Response to isoquinoline alkaloid	29/18862	0.030319691	0.093433678
BP	GO:0019048	Modulation by virus of host process	29/18862	0.030319691	0.093433678
BP	GO:0036296	Response to increased oxygen levels	29/18862	0.030319691	0.093433678
BP	GO:0043278	Response to morphine	29/18862	0.030319691	0.093433678
BP	GO:0019883	Antigen processing and presentation of endogenous antigen	26/18862	0.027224146	0.089057124
BP	GO:0036475	Neuron death in response to oxidative stress	26/18862	0.027224146	0.089057124
BP	GO:0044068	Modulation by symbiont of host cellular process	24/18862	2.03×10^{-6}	3.09×10^{-5}
BP	GO:0000423	Mitophagy	24/18862	0.000290711	0.001931699
BP	GO:0031668	Cellular response to extracellular stimulus	235/18862	9.73×10^{-5}	0.000795472
BP	GO:1901984	Negative regulation of protein acetylation	23/18862	0.024119214	0.081404865
BP	GO:0045732	Positive regulation of protein catabolic process	225/18862	0.023370866	0.080151356
BP	GO:0045061	Thymic T cell selection	22/18862	0.023082146	0.080151356
BP	GO:0050765	Negative regulation of phagocytosis	22/18862	0.023082146	0.080151356
BP	GO:0055093	Response to hyperoxia	22/18862	0.023082146	0.080151356
BP	GO:1903203	Regulation of oxidative stress-induced neuron death	22/18862	0.023082146	0.080151356
BP	GO:0051701	Biological process involved in interaction with host	219/18862	0.001521086	0.008741375
BP	GO:0031669	Cellular response to nutrient levels	210/18862	6.29×10^{-5}	0.000636865
BP	GO:0043984	Histone H4-K16 acetylation	21/18862	0.022044031	0.080151356
BP	GO:0098780	Response to mitochondrial depolarisation	20/18862	0.000200637	0.001471095
BP	GO:0042594	Response to starvation	196/18862	1.57×10^{-6}	2.57×10^{-5}
BP	GO:2000757	Negative regulation of peptidyl-lysine acetylation	19/18862	0.019964657	0.075805653
BP	GO:0019054	Modulation by virus of host cellular process	18/18862	0.018923396	0.07315839
BP	GO:0045721	Negative regulation of gluconeogenesis	18/18862	0.018923396	0.07315839
BP	GO:0007033	Vacuole organization	176/18862	3.52×10^{-20}	1.50×10^{-18}
BP	GO:2000104	Negative regulation of DNA-dependent DNA replication	17/18862	0.017881083	0.071737414
BP	GO:0016241	Regulation of macroautophagy	160/18862	0.000614164	0.003731159
BP	GO:0035067	Negative regulation of histone acetylation	16/18862	0.016837719	0.06885059
BP	GO:0061684	Chaperone-mediated autophagy	16/18862	0.016837719	0.06885059
BP	GO:1903204	Negative regulation of oxidative stress-induced neuron death	16/18862	0.016837719	0.06885059
BP	GO:0009267	Cellular response to starvation	157/18862	2.02×10^{-5}	0.000214479
BP	GO:0044804	Autophagy of nucleus	14/18862	9.50×10^{-22}	5.05×10^{-20}
BP	GO:0018410	C-terminal protein amino acid modification	14/18862	9.14×10^{-10}	1.94×10^{-8}
BP	GO:0036295	Cellular response to increased oxygen levels	14/18862	0.01474783	0.063997028
BP	GO:0090239	Regulation of histone H4 acetylation	14/18862	0.01474783	0.063997028

(*Continued*)

Table S3: *Continued*

Ontology	ID	Description	BgRatio	*p*value	*q*value
BP	GO:1901096	Regulation of autophagosome maturation	14/18862	0.01474783	0.063997028
BP	GO:0042157	Lipoprotein metabolic process	133/18862	1.05×10^{-5}	0.000117359
BP	GO:0006995	Cellular response to nitrogen starvation	13/18862	8.27×10^{-5}	0.000732997
BP	GO:0043562	Cellular response to nitrogen levels	13/18862	8.27×10^{-5}	0.000732997
BP	GO:0019079	Viral genome replication	129/18862	0.0003272	0.002108277
BP	GO:0035821	Modulation of process of other organism	123/18862	0.000284461	0.001931699
BP	GO:0061709	Reticulophagy	12/18862	7.01×10^{-5}	0.000677043
BP	GO:0006264	Mitochondrial DNA replication	12/18862	0.012653721	0.058490886
BP	GO:0043383	Negative T cell selection	12/18862	0.012653721	0.058490886
BP	GO:0055012	Ventricular cardiac muscle cell differentiation	12/18862	0.012653721	0.058490886
BP	GO:0034497	Protein localization to phagophore assembly site	11/18862	1.67×10^{-7}	3.23×10^{-6}
BP	GO:0045060	Negative thymic T cell selection	11/18862	0.011605082	0.057386208
BP	GO:0048102	Autophagic cell death	11/18862	0.011605082	0.057386208
BP	GO:0061635	Regulation of protein complex stability	11/18862	0.011605082	0.057386208
BP	GO:1903008	Organelle disassembly	107/18862	6.88×10^{-16}	1.83×10^{-14}
BP	GO:0098792	Xenophagy	10/18862	0.010555385	0.057386208
CC	GO:0005776	Autophagosome	98/19520	4.75×10^{-12}	2.33×10^{-11}
CC	GO:0005811	Lipid droplet	95/19520	0.004206033	0.003873978
CC	GO:0030670	Phagocytic vesicle membrane	77/19520	0.002786874	0.002933552
CC	GO:0005774	Vacuolar membrane	431/19520	6.04×10^{-5}	0.000100659
CC	GO:0000421	Autophagosome membrane	40/19520	7.15×10^{-8}	2.63×10^{-7}
CC	GO:0044232	Organelle membrane contact site	39/19520	0.000722418	0.000818936
CC	GO:0000407	Phagophore assembly site	31/19520	2.77×10^{-30}	4.08×10^{-29}
CC	GO:0030139	Endocytic vesicle	307/19520	0.003601341	0.003538159
CC	GO:0019898	Extrinsic component of membrane	304/19520	1.13×10^{-5}	2.39×10^{-5}
CC	GO:0005942	Phosphatidylinositol 3-kinase complex	29/19520	0.029311534	0.022734708
CC	GO:0099568	Cytoplasmic region	258/19520	0.000122273	0.000180192
CC	GO:0061695	Transferase complex, transferring phosphorus-containing groups	253/19520	0.02726677	0.022323671
CC	GO:0032838	Plasma membrane bounded cell projection cytoplasm	216/19520	6.15×10^{-5}	0.000100659
CC	GO:0044233	Mitochondria-associated endoplasmic reticulum membrane	20/19520	0.00018741	0.000251076
CC	GO:0030666	Endocytic vesicle membrane	163/19520	0.011930343	0.010342093
CC	GO:0034045	Phagophore assembly site membrane	15/19520	1.55×10^{-21}	1.14×10^{-20}
CC	GO:0045335	Phagocytic vesicle	136/19520	0.000345757	0.000424614
CC	GO:0097014	Ciliary plasm	130/19520	8.38×10^{-6}	2.06×10^{-5}
CC	GO:0005930	Axoneme	128/19520	7.88×10^{-6}	2.06×10^{-5}
MF	GO:0120013	Lipid transfer activity	46/18337	0.044224604	0.029624136
MF	GO:0005543	Phospholipid binding	451/18337	0.009161349	0.010289852
MF	GO:0032266	Phosphatidylinositol-3-phosphate binding	42/18337	8.90×10^{-6}	6.56×10^{-5}
MF	GO:0016877	Ligase activity, forming carbon-sulfur bonds	41/18337	0.039508616	0.029111612
MF	GO:0080025	Phosphatidylinositol-3,5-bisphosphate binding	27/18337	0.026186657	0.021439368

(*Continued*)

Table S3: *Continued*

Ontology	ID	Description	BgRatio	*p*value	*q*value
MF	GO:0035091	Phosphatidylinositol binding	253/18337	0.001816826	0.003346785
MF	GO:0051020	GTPase binding	222/18337	0.019651948	0.018100478
MF	GO:0008234	Cysteine-type peptidase activity	174/18337	0.000617033	0.00151552
MF	GO:1901981	Phosphatidylinositol phosphate binding	171/18337	0.000586565	0.00151552
MF	GO:0004197	Cysteine-type endopeptidase activity	117/18337	0.005776867	0.008513277
MF	GO:0008641	Ubiquitin-like modifier activating enzyme activity	10/18337	0.009775359	0.010289852

Table S4: Analysis of KEGG

ID	Description	BgRatio	*p*value	*q*value
hsa04140	Regulation of autophagy	34/5894	8.77×10^{-16}	2.77×10^{-15}
hsa04622	RIG-I-like receptor signaling pathway	71/5894	0.00382286	0.006036094

Table S6: SVM-RFE genes

ATG14	KIAA1324	DRAM1	KIAA1324L	ATG101
ATG7	EI24	ATG2B	ATG4B	KIAA0226
FYCO1	KIAA0226L	ATG16L2	WIPI2	ATG5
ATG12	SOGA1	ATG13		

Appendix 4

A4 LASSO and SVM-RFE genes

Table S5: LASSO genes

DRAM1	FYCO1	ATG14	KIAA1324	C10orf10
SOGA1	ATG7	KIAA0226	KIAA1324L	KIAA0226L
ATG16L2	ATG12			

Table S7: InterGenes

DRAM1	ATG16L2	ATG7	ATG14	KIAA1324
SOGA1	FYCO1	ATG12	KIAA0226	KIAA1324L
KIAA0226L				

Appendix 5

A5 GSEA analysis

Table S8: ATG16L2 of GSEA analysis

Description	enrichmentScore	*p*value	*q*value	rank
KEGG_UBIQUITIN_MEDIATED_PROTEOLYSIS	0.512633831	1.99×10^{-8}	2.49×10^{-6}	4770
KEGG_NOD_LIKE_RECEPTOR_SIGNALING_PATHWAY	0.620142631	4.44×10^{-8}	2.78×10^{-6}	4539
KEGG_CYTOKINE_CYTOKINE_RECEPTOR_INTERACTION	0.409806857	2.22×10^{-7}	9.26×10^{-6}	3197
KEGG_TOLL_LIKE_RECEPTOR_SIGNALING_PATHWAY	0.520300685	4.04×10^{-7}	1.27×10^{-5}	4457
KEGG_LEISHMANIA_INFECTION	0.5725801	8.81×10^{-7}	2.21×10^{-5}	4628
KEGG_SPLICEOSOME	0.481251175	1.56×10^{-6}	3.26×10^{-5}	6347
KEGG_APOPTOSIS	0.517581305	2.11×10^{-6}	3.78×10^{-5}	5711
KEGG_CHEMOKINE_SIGNALING_PATHWAY	0.423616518	4.57×10^{-6}	7.15×10^{-5}	4626
KEGG_T_CELL_RECEPTOR_SIGNALING_PATHWAY	0.477203483	6.65×10^{-6}	9.26×10^{-5}	6270
KEGG_LYSOSOME	0.458107509	8.66×10^{-6}	0.000108509	5257
KEGG_OLFACTORY_TRANSDUCTION	−0.412405851	3.24×10^{-5}	0.000368594	5746
KEGG_PRIMARY_IMMUNODEFICIENCY	0.619044818	4.49×10^{-5}	0.000453456	3032
KEGG_DRUG_METABOLISM_CYTOCHROME_P450	−0.486298613	4.71×10^{-5}	0.000453456	3544
KEGG_NEUROACTIVE_LIGAND_RECEPTOR_INTERACTION	−0.302824513	7.23×10^{-5}	0.000646645	4728
KEGG_ALLOGRAFT_REJECTION	0.610681516	0.000138486	0.00115648	3032
KEGG_PROTEASOME	0.573209282	0.000214431	0.001475984	5880
KEGG_TYPE_I_DIABETES_MELLITUS	0.572210706	0.000190431	0.001475984	2914
KEGG_SYSTEMIC_LUPUS_ERYTHEMATOSUS	0.528413716	0.000218226	0.001475984	5320
KEGG_B_CELL_RECEPTOR_SIGNALING_PATHWAY	0.472386835	0.000223878	0.001475984	6137
KEGG_NATURAL_KILLER_CELL_MEDIATED_CYTOTOXICITY	0.400500386	0.000407159	0.002550103	3271
KEGG_METABOLISM_OF_XENOBIOTICS_BY_CYTOCHROME_P450	−0.453081102	0.000502084	0.002994885	5216
KEGG_RENAL_CELL_CARCINOMA	0.46604456	0.000581933	0.003313399	7698
KEGG_GRAFT_VERSUS_HOST_DISEASE	0.567371304	0.000671035	0.00350233	2914
KEGG_JAK_STAT_SIGNALING_PATHWAY	0.3798737	0.00065953	0.00350233	4621
KEGG_HEMATOPOIETIC_CELL_LINEAGE	0.438523748	0.000866874	0.004343497	5314
KEGG_PENTOSE_PHOSPHATE_PATHWAY	0.593274121	0.001072519	0.005167196	6286
KEGG_RNA_DEGRADATION	0.48630997	0.001192288	0.005531471	7299
KEGG_SMALL_CELL_LUNG_CANCER	0.431101496	0.001243238	0.005561856	5339
KEGG_DILATED_CARDIOMYOPATHY	−0.381313395	0.00164103	0.007088299	4463
KEGG_CALCIUM_SIGNALING_PATHWAY	−0.302181472	0.002090687	0.008729536	4475
KEGG_FC_GAMMA_R_MEDIATED_PHAGOCYTOSIS	0.414917005	0.002275739	0.008936976	3611
KEGG_CELL_CYCLE	0.384440269	0.002283059	0.008936976	6187
KEGG_CELL_ADHESION_MOLECULES_CAMS	0.378313299	0.002481015	0.00941757	3336
KEGG_CYTOSOLIC_DNA_SENSING_PATHWAY	0.464328584	0.003499166	0.012891666	4856

(*Continued*)

Table S8: *Continued*

Description	enrichmentScore	*p*value	*q*value	rank
KEGG_ECM_RECEPTOR_INTERACTION	0.413902884	0.003843812	0.013756802	3255
KEGG_CARDIAC_MUSCLE_CONTRACTION	−0.374686151	0.004455607	0.015503427	3556
KEGG_NEUROTROPHIN_SIGNALING_PATHWAY	0.368173842	0.005620962	0.019029716	6171
KEGG_PPAR_SIGNALING_PATHWAY	0.41372368	0.006783049	0.022359635	2828
KEGG_SPHINGOLIPID_METABOLISM	0.516122022	0.00808274	0.02469438	2460
KEGG_AUTOIMMUNE_THYROID_DISEASE	0.451146141	0.007706807	0.02469438	3175
KEGG_HYPERTROPHIC_CARDIOMYOPATHY_HCM	−0.352646374	0.008067614	0.02469438	7028
KEGG_ETHER_LIPID_METABOLISM	0.567261027	0.009140146	0.027260084	1596
KEGG_BASAL_TRANSCRIPTION_FACTORS	0.516954324	0.009651471	0.028115669	5258
KEGG_P53_SIGNALING_PATHWAY	0.407921495	0.009905275	0.028199227	4496
KEGG_STEROID_BIOSYNTHESIS	0.621939903	0.011550426	0.030982789	2669
KEGG_CITRATE_CYCLE_TCA_CYCLE	0.509007466	0.011203385	0.030982789	6944
KEGG_RETINOL_METABOLISM	−0.408976691	0.011625055	0.030982789	3881
KEGG_PANCREATIC_CANCER	0.401766748	0.011935623	0.031147788	6970
KEGG_SNARE_INTERACTIONS_IN_VESICULAR_TRANSPORT	0.479844498	0.013682157	0.034976942	5467
KEGG_CHRONIC_MYELOID_LEUKEMIA	0.388438526	0.017280255	0.043291586	6970
KEGG_OXIDATIVE_PHOSPHORYLATION	0.358480186	0.019557225	0.04803529	5089
KEGG_BETA_ALANINE_METABOLISM	0.515765562	0.024039223	0.056351427	2724
KEGG_ENDOCYTOSIS	0.308216091	0.023806771	0.056351427	4486
KEGG_PATHWAYS_IN_CANCER	0.272108677	0.024292674	0.056351427	5708
KEGG_FATTY_ACID_METABOLISM	0.447002178	0.02589599	0.058978427	2895
KEGG_INTESTINAL_IMMUNE_NETWORK_FOR_IGA_PRODUCTION	0.440615935	0.032362782	0.071120427	4242
KEGG_EPITHELIAL_CELL_SIGNALING_IN_HELICOBACTER_PYLORI_INFECTION	0.386621147	0.032013867	0.071120427	4856
KEGG_ANTIGEN_PROCESSING_AND_PRESENTATION	0.362212673	0.039513678	0.08533807	4742
KEGG_MELANOGENESIS	−0.294193019	0.042320074	0.089849935	5068
KEGG_TRYPTOPHAN_METABOLISM	0.413560986	0.044067797	0.091331888	2724
KEGG_ALZHEIMERS_DISEASE	0.31500864	0.044476327	0.091331888	7182
KEGG_PROPANOATE_METABOLISM	0.455077971	0.0458125	0.092558362	2724
KEGG_GLYCOSAMINOGLYCAN_DEGRADATION	0.50783904	0.048561151	0.094826811	2599
KEGG_COLORECTAL_CANCER	0.369103486	0.048309179	0.094826811	7137
KEGG_VEGF_SIGNALING_PATHWAY	0.355962475	0.049206349	0.094826811	5711

Table S9: DRAM1 of GSEA analysis

ID	enrichmentScore	*p*value	*q*value	rank
KEGG_UBIQUITIN_MEDIATED_PROTEOLYSIS	0.542923972	1.00×10^{-10}	1.22×10^{-8}	4111
KEGG_NEUROACTIVE_LIGAND_RECEPTOR_INTERACTION	−0.363730886	1.31×10^{-9}	8.00×10^{-8}	6023
KEGG_SPLICEOSOME	0.51766829	6.23×10^{-9}	2.54×10^{-7}	7244
KEGG_NOD_LIKE_RECEPTOR_SIGNALING_PATHWAY	0.552868753	1.73×10^{-6}	4.99×10^{-5}	5875
KEGG_TOLL_LIKE_RECEPTOR_SIGNALING_PATHWAY	0.490284354	2.05×10^{-6}	4.99×10^{-5}	5360
KEGG_LEISHMANIA_INFECTION	0.527388277	5.01×10^{-6}	8.74×10^{-5}	6890
KEGG_OLFACTORY_TRANSDUCTION	−0.413544747	4.95×10^{-6}	8.74×10^{-5}	6187
KEGG_RNA_DEGRADATION	0.543203163	5.92×10^{-6}	9.03×10^{-5}	7244
KEGG_T_CELL_RECEPTOR_SIGNALING_PATHWAY	0.457574845	6.85×10^{-6}	9.29×10^{-5}	6041
KEGG_PROTEASOME	0.590422883	1.59×10^{-5}	0.000194198	6693
KEGG_CELL_CYCLE	0.432184042	2.58×10^{-5}	0.00028666	5890
KEGG_LYSOSOME	0.431726472	3.60×10^{-5}	0.000366559	7338
KEGG_RENAL_CELL_CARCINOMA	0.492127331	6.05×10^{-5}	0.000567987	5447
KEGG_APOPTOSIS	0.449954736	7.29×10^{-5}	0.000594534	5493
KEGG_CALCIUM_SIGNALING_PATHWAY	−0.33037581	7.30×10^{-5}	0.000594534	5251
KEGG_P53_SIGNALING_PATHWAY	0.474888661	0.000193785	0.001478885	5985
KEGG_FC_GAMMA_R_MEDIATED_PHAGOCYTOSIS	0.423482559	0.000250146	0.001796716	5826
KEGG_B_CELL_RECEPTOR_SIGNALING_PATHWAY	0.451279852	0.000467935	0.003163329	7012
KEGG_CHEMOKINE_SIGNALING_PATHWAY	0.347928702	0.000492225	0.003163329	5447
KEGG_CHRONIC_MYELOID_LEUKEMIA	0.438385376	0.000621389	0.003793744	5648
KEGG_PATHOGENIC_ESCHERICHIA_COLI_INFECTION	0.48398935	0.000739363	0.004299055	6899
KEGG_PARKINSONS_DISEASE	0.422726909	0.000782967	0.004314165	7432
KEGG_PANCREATIC_CANCER	0.442858552	0.000812625	0.004314165	7012
KEGG_CYTOKINE_CYTOKINE_RECEPTOR_INTERACTION	0.312896676	0.000948216	0.004824255	3656
KEGG_CELL_ADHESION_MOLECULES_CAMS	0.376986332	0.001042112	0.005089892	2291
KEGG_CYTOSOLIC_DNA_SENSING_PATHWAY	0.467787869	0.001280409	0.005984963	4952
KEGG_ENDOCYTOSIS	0.345135015	0.001323399	0.005984963	5536
KEGG_PROTEIN_EXPORT	0.586543249	0.001597607	0.006502541	7696
KEGG_SPHINGOLIPID_METABOLISM	0.534015836	0.001510292	0.006502541	2134
KEGG_SMALL_CELL_LUNG_CANCER	0.409538969	0.001560884	0.006502541	5405
KEGG_PYRIMIDINE_METABOLISM	0.396665747	0.002004063	0.007893762	4589
KEGG_OXIDATIVE_PHOSPHORYLATION	0.387303128	0.002683868	0.010241074	7432
KEGG_HEDGEHOG_SIGNALING_PATHWAY	−0.403881537	0.003208221	0.011870928	5523
KEGG_BASAL_TRANSCRIPTION_FACTORS	0.51078633	0.003832299	0.013763055	5659
KEGG_MATURITY_ONSET_DIABETES_OF_THE_YOUNG	−0.526561946	0.004326431	0.014674444	3604
KEGG_FOCAL_ADHESION	0.313404043	0.004238963	0.014674444	5779
KEGG_EPITHELIAL_CELL_SIGNALING_IN_HELICOBACTER_PYLORI_INFECTION	0.408386372	0.004657624	0.015222788	7014
KEGG_DILATED_CARDIOMYOPATHY	−0.340085532	0.004737437	0.015222788	6821
KEGG_SNARE_INTERACTIONS_IN_VESICULAR_TRANSPORT	0.476264886	0.00514879	0.015717358	6207
KEGG_COLORECTAL_CANCER	0.417883707	0.005024147	0.015717358	7189

(*Continued*)

Table S9: *Continued*

ID	enrichmentScore	*p*value	*q*value	rank
KEGG_NEUROTROPHIN_SIGNALING_PATHWAY	0.353983166	0.005931833	0.017666048	5672
KEGG_BLADDER_CANCER	0.469897339	0.006475289	0.018825403	5648
KEGG_PRIMARY_IMMUNODEFICIENCY	0.477295999	0.006639607	0.018854209	5181
KEGG_AMINO_SUGAR_AND_NUCLEOTIDE_SUGAR_METABOLISM	0.455039556	0.006985763	0.019386327	1257
KEGG_BETA_ALANINE_METABOLISM	0.534926284	0.007581495	0.020572011	2268
KEGG_ALZHEIMERS_DISEASE	0.339213531	0.008300054	0.022032181	6302
KEGG_ENDOMETRIAL_CANCER	0.421823204	0.009433147	0.024507169	7178
KEGG_PATHWAYS_IN_CANCER	0.279296293	0.010141376	0.025798236	5648
KEGG_CITRATE_CYCLE_TCA_CYCLE	0.483423286	0.010624849	0.026060331	6510
KEGG_NATURAL_KILLER_CELL_MEDIATED_CYTOTOXICITY	0.329119094	0.010671256	0.026060331	5332
KEGG_TASTE_TRANSDUCTION	−0.390337837	0.012029112	0.028800352	3526
KEGG_HYPERTROPHIC_CARDIOMYOPATHY_HCM	−0.322268344	0.015358095	0.036063543	8020
KEGG_ECM_RECEPTOR_INTERACTION	0.359065092	0.017975219	0.040983709	5309
KEGG_JAK_STAT_SIGNALING_PATHWAY	0.307676795	0.018124692	0.040983709	5287
KEGG_GRAFT_VERSUS_HOST_DISEASE	0.432933711	0.019221254	0.042598889	2772
KEGG_CARDIAC_MUSCLE_CONTRACTION	−0.328432923	0.019536732	0.042598889	2931
KEGG_PENTOSE_PHOSPHATE_PATHWAY	0.484162172	0.020861088	0.044688572	4225
KEGG_TYPE_I_DIABETES_MELLITUS	0.431973734	0.022345479	0.047043114	5835
KEGG_PROPANOATE_METABOLISM	0.452772839	0.024150154	0.049980694	2268
KEGG_HEMATOPOIETIC_CELL_LINEAGE	0.3450755	0.032839405	0.066831069	3656
KEGG_ACUTE_MYELOID_LEUKEMIA	0.386789796	0.03869969	0.076216708	5880
KEGG_OOCYTE_MEIOSIS	0.322911006	0.038461538	0.076216708	5773
KEGG_NUCLEOTIDE_EXCISION_REPAIR	0.398553508	0.040498442	0.077266765	2498
KEGG_PEROXISOME	0.352632331	0.040198259	0.077266765	2713
KEGG_ADHERENS_JUNCTION	0.357852935	0.042748092	0.080304107	6868
KEGG_ALLOGRAFT_REJECTION	0.438204267	0.046624697	0.084971953	7690
KEGG_ETHER_LIPID_METABOLISM	0.459803477	0.046589018	0.084971953	1003
KEGG_TRYPTOPHAN_METABOLISM	0.39824366	0.047923323	0.086054264	2268

Appendix 6

A6 MiRNA and LncRNA

Table S10: Gene-miRNA

Gene	miRNA	miRanda	miRDB	TargetScan	Sum
FYCO1	hsa-miR-4288	1	1	1	3
KIAA1324L	hsa-miR-541-3p	1	1	1	3
ATG7	hsa-miR-149-5p	1	1	1	3
KIAA1324	hsa-miR-302b-5p	1	1	1	3
KIAA1324L	hsa-miR-148b-3p	1	1	1	3
FYCO1	hsa-miR-2115-3p	1	1	1	3
DRAM1	hsa-miR-576-3p	1	1	1	3
KIAA1324	hsa-miR-30b-3p	1	1	1	3
FYCO1	hsa-miR-182-3p	1	1	1	3
KIAA1324L	hsa-miR-3163	1	1	1	3
ATG7	hsa-miR-93-5p	1	1	1	3
DRAM1	hsa-miR-4282	1	1	1	3
KIAA1324L	hsa-miR-4282	1	1	1	3
KIAA1324L	hsa-miR-3133	1	1	1	3
KIAA1324L	hsa-miR-4291	1	1	1	3
FYCO1	hsa-miR-30b-5p	1	1	1	3
ATG16L2	hsa-miR-30c-2-3p	1	1	1	3
KIAA1324	hsa-miR-2110	1	1	1	3
DRAM1	hsa-miR-759	1	1	1	3
FYCO1	hsa-miR-3163	1	1	1	3
DRAM1	hsa-miR-26b-5p	1	1	1	3
DRAM1	hsa-miR-205-5p	1	1	1	3
ATG12	hsa-miR-4284	1	1	1	3
ATG7	hsa-miR-497-3p	1	1	1	3
ATG7	hsa-miR-486-3p	1	1	1	3
ATG7	hsa-miR-96-5p	1	1	1	3
FYCO1	hsa-miR-298	1	1	1	3
KIAA1324L	hsa-miR-561-3p	1	1	1	3
KIAA1324	hsa-miR-1200	1	1	1	3
DRAM1	hsa-miR-181a-5p	1	1	1	3
FYCO1	hsa-miR-485-5p	1	1	1	3
ATG7	hsa-miR-320b	1	1	1	3
KIAA1324	hsa-miR-3175	1	1	1	3
KIAA1324	hsa-miR-4307	1	1	1	3

(*Continued*)

Table S10: *Continued*

Gene	miRNA	miRanda	miRDB	TargetScan	Sum
KIAA1324L	hsa-miR-660-5p	1	1	1	3
ATG7	hsa-miR-106a-5p	1	1	1	3
ATG12	hsa-miR-1184	1	1	1	3
DRAM1	hsa-miR-3137	1	1	1	3
ATG12	hsa-miR-539-5p	1	1	1	3
KIAA1324	hsa-miR-609	1	1	1	3
DRAM1	hsa-miR-181b-5p	1	1	1	3
ATG7	hsa-miR-509-3p	1	1	1	3
FYCO1	hsa-miR-3166	1	1	1	3
KIAA1324	hsa-miR-485-5p	1	1	1	3
ATG12	hsa-miR-513a-3p	1	1	1	3
KIAA1324L	hsa-miR-92a-2-5p	1	1	1	3
KIAA1324L	hsa-miR-1236-3p	1	1	1	3
KIAA1324L	hsa-miR-186-3p	1	1	1	3
KIAA1324	hsa-miR-18a-3p	1	1	1	3
KIAA1324	hsa-miR-939-5p	1	1	1	3
FYCO1	hsa-miR-302d-3p	1	1	1	3
FYCO1	hsa-miR-30d-5p	1	1	1	3
ATG12	hsa-miR-3117-3p	1	1	1	3
ATG7	hsa-miR-548x-3p	1	1	1	3
KIAA1324L	hsa-miR-186-5p	1	1	1	3
FYCO1	hsa-miR-1200	1	1	1	3
ATG7	hsa-miR-4288	1	1	1	3
ATG7	hsa-miR-20b-5p	1	1	1	3
DRAM1	hsa-miR-195-5p	1	1	1	3
DRAM1	hsa-miR-4255	1	1	1	3
FYCO1	hsa-miR-544b	1	1	1	3
DRAM1	hsa-miR-4316	1	1	1	3
FYCO1	hsa-miR-4267	1	1	1	3
DRAM1	hsa-miR-181c-5p	1	1	1	3
KIAA1324L	hsa-miR-548c-3p	1	1	1	3
DRAM1	hsa-miR-4261	1	1	1	3
KIAA1324	hsa-miR-1225-5p	1	1	1	3
FYCO1	hsa-miR-632	1	1	1	3
ATG7	hsa-miR-20b-3p	1	1	1	3
DRAM1	hsa-miR-582-3p	1	1	1	3
ATG12	hsa-miR-1323	1	1	1	3
DRAM1	hsa-miR-548c-3p	1	1	1	3
ATG7	hsa-miR-103b	1	1	1	3
FYCO1	hsa-miR-665	1	1	1	3

(*Continued*)

Table S10: *Continued*

Gene	miRNA	miRanda	miRDB	TargetScan	Sum
ATG12	hsa-miR-548c-3p	1	1	1	3
KIAA1324L	hsa-miR-3065-5p	1	1	1	3
FYCO1	hsa-miR-142-3p	1	1	1	3
FYCO1	hsa-miR-4297	1	1	1	3
ATG12	hsa-miR-187-5p	1	1	1	3
KIAA1324	hsa-miR-544b	1	1	1	3
FYCO1	hsa-miR-2861	1	1	1	3
ATG7	hsa-miR-1205	1	1	1	3
ATG7	hsa-miR-582-5p	1	1	1	3
FYCO1	hsa-miR-194-3p	1	1	1	3
FYCO1	hsa-miR-4264	1	1	1	3
FYCO1	hsa-miR-4276	1	1	1	3
ATG7	hsa-miR-765	1	1	1	3
ATG12	hsa-miR-944	1	1	1	3
KIAA1324	hsa-miR-3125	1	1	1	3
DRAM1	hsa-miR-524-5p	1	1	1	3
FYCO1	hsa-miR-520d-3p	1	1	1	3
KIAA1324	hsa-miR-4276	1	1	1	3
KIAA1324	hsa-miR-330-3p	1	1	1	3
ATG12	hsa-miR-633	1	1	1	3
DRAM1	hsa-miR-3153	1	1	1	3
FYCO1	hsa-miR-1915-3p	1	1	1	3
FYCO1	hsa-miR-486-3p	1	1	1	3
KIAA1324	hsa-miR-320d	1	1	1	3
FYCO1	hsa-miR-1233-3p	1	1	1	3
DRAM1	hsa-miR-650	1	1	1	3
KIAA1324	hsa-miR-3163	1	1	1	3
FYCO1	hsa-miR-3125	1	1	1	3
KIAA1324L	hsa-miR-1200	1	1	1	3
FYCO1	hsa-miR-1914-5p	1	1	1	3
KIAA1324L	hsa-miR-452-5p	1	1	1	3
DRAM1	hsa-miR-877-3p	1	1	1	3
DRAM1	hsa-miR-588	1	1	1	3
ATG7	hsa-miR-569	1	1	1	3
FYCO1	hsa-miR-636	1	1	1	3
ATG12	hsa-miR-630	1	1	1	3
KIAA1324L	hsa-miR-3161	1	1	1	3
ATG12	hsa-let-7f-1-3p	1	1	1	3
ATG16L2	hsa-miR-524-5p	1	1	1	3
KIAA1324	hsa-miR-373-5p	1	1	1	3

(*Continued*)

Table S10: *Continued*

Gene	miRNA	miRanda	miRDB	TargetScan	Sum
KIAA1324	hsa-miR-1304-5p	1	1	1	3
ATG12	hsa-miR-4286	1	1	1	3
FYCO1	hsa-miR-1271-5p	1	1	1	3
ATG12	hsa-miR-545-5p	1	1	1	3
KIAA1324L	hsa-miR-944	1	1	1	3
KIAA1324L	hsa-miR-3144-3p	1	1	1	3
FYCO1	hsa-miR-526b-5p	1	1	1	3
DRAM1	hsa-miR-223-5p	1	1	1	3
ATG12	hsa-miR-2113	1	1	1	3
ATG12	hsa-miR-198	1	1	1	3
KIAA1324L	hsa-miR-551b-5p	1	1	1	3
KIAA1324	hsa-miR-938	1	1	1	3
ATG7	hsa-miR-181c-3p	1	1	1	3
KIAA1324	hsa-miR-495-3p	1	1	1	3
FYCO1	hsa-miR-93-5p	1	1	1	3
KIAA1324	hsa-miR-665	1	1	1	3
ATG12	hsa-miR-548t-5p	1	1	1	3
KIAA1324	hsa-miR-1913	1	1	1	3
FYCO1	hsa-miR-2116-5p	1	1	1	3
KIAA1324L	hsa-miR-590-3p	1	1	1	3
FYCO1	hsa-miR-92a-2-5p	1	1	1	3
KIAA1324	hsa-miR-921	1	1	1	3
ATG12	hsa-miR-4272	1	1	1	3
KIAA1324L	hsa-miR-1178-3p	1	1	1	3
DRAM1	hsa-miR-136-5p	1	1	1	3
KIAA1324	hsa-miR-1202	1	1	1	3
ATG7	hsa-miR-1207-5p	1	1	1	3
FYCO1	hsa-miR-130a-3p	1	1	1	3
ATG7	hsa-miR-632	1	1	1	3
ATG7	hsa-miR-1271-5p	1	1	1	3
DRAM1	hsa-miR-1262	1	1	1	3
FYCO1	hsa-miR-218-1-3p	1	1	1	3
KIAA1324	hsa-miR-320b	1	1	1	3
KIAA1324L	hsa-miR-3123	1	1	1	3
FYCO1	hsa-miR-513a-3p	1	1	1	3
FYCO1	hsa-miR-302b-3p	1	1	1	3
FYCO1	hsa-miR-3126-5p	1	1	1	3
DRAM1	hsa-miR-569	1	1	1	3
ATG7	hsa-miR-708-3p	1	1	1	3
ATG7	hsa-miR-3137	1	1	1	3

(*Continued*)

Table S10: *Continued*

Gene	miRNA	miRanda	miRDB	TargetScan	Sum
KIAA1324L	hsa-miR-27a-5p	1	1	1	3
ATG12	hsa-miR-761	1	1	1	3
ATG12	hsa-miR-586	1	1	1	3
FYCO1	hsa-miR-106b-5p	1	1	1	3
FYCO1	hsa-miR-1224-5p	1	1	1	3
FYCO1	hsa-miR-590-3p	1	1	1	3
FYCO1	hsa-miR-4271	1	1	1	3
FYCO1	hsa-miR-186-5p	1	1	1	3
FYCO1	hsa-miR-1253	1	1	1	3
ATG7	hsa-miR-190a-5p	1	1	1	3
KIAA1324L	hsa-miR-455-5p	1	1	1	3
FYCO1	hsa-miR-377-5p	1	1	1	3
ATG7	hsa-miR-647	1	1	1	3
KIAA1324L	hsa-miR-4324	1	1	1	3
ATG12	hsa-miR-19a-5p	1	1	1	3
DRAM1	hsa-miR-1297	1	1	1	3
DRAM1	hsa-miR-4294	1	1	1	3
ATG12	hsa-miR-4307	1	1	1	3
ATG7	hsa-miR-612	1	1	1	3
FYCO1	hsa-miR-542-3p	1	1	1	3
DRAM1	hsa-miR-3163	1	1	1	3
FYCO1	hsa-miR-9-3p	1	1	1	3
ATG7	hsa-miR-1285-3p	1	1	1	3
FYCO1	hsa-miR-4282	1	1	1	3
KIAA1324	hsa-miR-320c	1	1	1	3
KIAA1324	hsa-miR-31-3p	1	1	1	3
ATG7	hsa-miR-302e	1	1	1	3
DRAM1	hsa-miR-2054	1	1	1	3
KIAA1324L	hsa-miR-571	1	1	1	3
FYCO1	hsa-miR-96-5p	1	1	1	3
KIAA1324L	hsa-miR-542-3p	1	1	1	3
ATG12	hsa-miR-570-3p	1	1	1	3
ATG12	hsa-miR-3163	1	1	1	3
ATG12	hsa-miR-2355-5p	1	1	1	3
FYCO1	hsa-miR-301b-3p	1	1	1	3
KIAA1324	hsa-miR-1283	1	1	1	3
FYCO1	hsa-miR-106a-5p	1	1	1	3
KIAA1324L	hsa-miR-182-5p	1	1	1	3
FYCO1	hsa-miR-214-5p	1	1	1	3
DRAM1	hsa-miR-324-5p	1	1	1	3

(*Continued*)

Table S10: *Continued*

Gene	miRNA	miRanda	miRDB	TargetScan	Sum
FYCO1	hsa-miR-182-5p	1	1	1	3
KIAA1324L	hsa-miR-3148	1	1	1	3
ATG7	hsa-miR-1267	1	1	1	3
FYCO1	hsa-miR-1205	1	1	1	3
FYCO1	hsa-miR-302a-3p	1	1	1	3
KIAA1324L	hsa-miR-31-5p	1	1	1	3
FYCO1	hsa-miR-1972	1	1	1	3
FYCO1	hsa-miR-1184	1	1	1	3
ATG12	hsa-miR-1305	1	1	1	3
KIAA1324L	hsa-miR-148a-3p	1	1	1	3
ATG12	hsa-miR-2052	1	1	1	3
ATG7	hsa-miR-223-3p	1	1	1	3
FYCO1	hsa-miR-7-5p	1	1	1	3
ATG7	hsa-miR-129-5p	1	1	1	3
ATG12	hsa-miR-802	1	1	1	3
KIAA1324	hsa-miR-548t-5p	1	1	1	3
ATG12	hsa-miR-214-3p	1	1	1	3
FYCO1	hsa-miR-130b-3p	1	1	1	3
ATG12	hsa-miR-4251	1	1	1	3
FYCO1	hsa-miR-520a-3p	1	1	1	3
DRAM1	hsa-miR-424-5p	1	1	1	3
FYCO1	hsa-miR-373-3p	1	1	1	3
ATG7	hsa-miR-4267	1	1	1	3
FYCO1	hsa-miR-765	1	1	1	3
DRAM1	hsa-miR-497-5p	1	1	1	3
KIAA1324L	hsa-miR-506-3p	1	1	1	3
FYCO1	hsa-miR-9-5p	1	1	1	3
FYCO1	hsa-miR-545-5p	1	1	1	3
DRAM1	hsa-miR-26a-5p	1	1	1	3
KIAA1324L	hsa-miR-4297	1	1	1	3
FYCO1	hsa-miR-548a-3p	1	1	1	3
ATG7	hsa-miR-483-3p	1	1	1	3
FYCO1	hsa-miR-573	1	1	1	3
FYCO1	hsa-miR-218-5p	1	1	1	3
ATG7	hsa-miR-331-5p	1	1	1	3
FYCO1	hsa-miR-4324	1	1	1	3
ATG12	hsa-miR-373-5p	1	1	1	3
KIAA1324L	hsa-miR-922	1	1	1	3
FYCO1	hsa-miR-659-3p	1	1	1	3
KIAA1324	hsa-miR-568	1	1	1	3

(*Continued*)

Table S10: *Continued*

Gene	miRNA	miRanda	miRDB	TargetScan	Sum
FYCO1	hsa-miR-1976	1	1	1	3
KIAA1324L	hsa-miR-30d-3p	1	1	1	3
ATG12	hsa-miR-18a-3p	1	1	1	3
ATG7	hsa-miR-106b-5p	1	1	1	3
KIAA1324L	hsa-let-7f-2-3p	1	1	1	3
DRAM1	hsa-miR-18a-3p	1	1	1	3
FYCO1	hsa-miR-548t-5p	1	1	1	3
KIAA1324	hsa-miR-302d-5p	1	1	1	3
KIAA1324L	hsa-miR-654-5p	1	1	1	3
ATG12	hsa-miR-1258	1	1	1	3
FYCO1	hsa-miR-138-5p	1	1	1	3
FYCO1	hsa-miR-20b-5p	1	1	1	3
ATG12	hsa-miR-1827	1	1	1	3
ATG12	hsa-miR-335-3p	1	1	1	3
DRAM1	hsa-miR-1827	1	1	1	3
DRAM1	hsa-let-7f-2-3p	1	1	1	3
ATG12	hsa-miR-1236-3p	1	1	1	3
FYCO1	hsa-miR-302e	1	1	1	3
ATG12	hsa-miR-4311	1	1	1	3
ATG7	hsa-miR-141-3p	1	1	1	3
FYCO1	hsa-miR-4253	1	1	1	3
DRAM1	hsa-miR-4276	1	1	1	3
FYCO1	hsa-miR-520c-3p	1	1	1	3
ATG7	hsa-miR-200a-3p	1	1	1	3
ATG7	hsa-miR-125b-2-3p	1	1	1	3
KIAA1324	hsa-miR-524-5p	1	1	1	3
KIAA1324	hsa-miR-556-5p	1	1	1	3
KIAA1324L	hsa-miR-4305	1	1	1	3
DRAM1	hsa-miR-587	1	1	1	3
ATG12	hsa-miR-4297	1	1	1	3
FYCO1	hsa-miR-4314	1	1	1	3
KIAA1324L	hsa-miR-1305	1	1	1	3
FYCO1	hsa-miR-30c-5p	1	1	1	3
KIAA1324L	hsa-miR-548t-5p	1	1	1	3
ATG7	hsa-miR-548t-5p	1	1	1	3
ATG7	hsa-miR-922	1	1	1	3
KIAA1324L	hsa-miR-591	1	1	1	3
DRAM1	hsa-miR-342-3p	1	1	1	3
ATG7	hsa-miR-20a-3p	1	1	1	3
ATG12	hsa-miR-548d-3p	1	1	1	3

(*Continued*)

Table S10: *Continued*

Gene	miRNA	miRanda	miRDB	TargetScan	Sum
ATG12	hsa-miR-3157-5p	1	1	1	3
ATG7	hsa-miR-1915-3p	1	1	1	3
DRAM1	hsa-miR-2053	1	1	1	3
DRAM1	hsa-miR-496	1	1	1	3
ATG16L2	hsa-miR-30b-3p	1	1	1	3
ATG12	hsa-miR-1272	1	1	1	3
ATG12	hsa-miR-3169	1	1	1	3
DRAM1	hsa-miR-16-5p	1	1	1	3
KIAA1324L	hsa-miR-548n	1	1	1	3
ATG7	hsa-miR-449b-3p	1	1	1	3
FYCO1	hsa-miR-29a-5p	1	1	1	3
ATG7	hsa-miR-4314	1	1	1	3
DRAM1	hsa-miR-139-5p	1	1	1	3
KIAA1324L	hsa-miR-544b	1	1	1	3
KIAA1324	hsa-miR-1827	1	1	1	3

Table S11: Gene-lncRNA

miRNA	lncRNA
hsa-miR-7-5p	CDR1-AS
hsa-miR-802	CTA-414D7.1
hsa-miR-1200	LINC01043
hsa-miR-765	GAS6-AS1
hsa-miR-612	RP11-326C3.10
hsa-miR-570-3p	RP11-10J21.4
hsa-miR-541-3p	RP11-10J21.4
hsa-miR-1202	HP09025
hsa-miR-541-3p	RP11-573D15.8
hsa-miR-214-3p	LA16c-306A4.2
hsa-miR-141-3p	RP11-830F9.6
hsa-miR-7-5p	RP11-830F9.6
hsa-miR-541-3p	C10orf91
hsa-miR-665	RP5-894D12.5
hsa-miR-612	RP1-34P24.3
hsa-miR-218-5p	RP5-894D12.5
hsa-miR-31-5p	C10orf91
hsa-miR-30b-3p	C10orf91
hsa-miR-198	MUC19
hsa-miR-1972	AC079779.7
hsa-miR-223-5p	AC069257.8
hsa-miR-136-5p	CTD-2534I21.9
hsa-miR-149-5p	LINC01043
hsa-miR-612	RP11-326C3.14
hsa-miR-148a-3p	CITF22-1A6.3
hsa-miR-130a-3p	CITF22-1A6.3
hsa-miR-181c-3p	CTD-2534I21.9
hsa-miR-7-5p	FLJ35934
hsa-miR-612	FAM230B
hsa-miR-486-3p	C10orf91
hsa-miR-921	CTA-722E9.1
hsa-miR-342-3p	LL22NC03-27C5.1
hsa-miR-1972	RP11-102K13.5
hsa-miR-665	RP11-627G23.1
hsa-miR-1972	RP5-894D12.5
hsa-miR-1972	RP13-580B18.4
hsa-miR-765	RP11-138B4.1
hsa-miR-541-3p	HP09025
hsa-miR-138-5p	HP09025
hsa-miR-939-5p	AATBC

(*Continued*)

Table S11: *Continued*

miRNA	lncRNA
hsa-miR-186-5p	RP11-99L13.2
hsa-miR-541-3p	PAX8-AS1
hsa-miR-541-3p	MUC2
hsa-miR-650	RP11-138B4.1
hsa-miR-223-5p	RP3-323A16.1
hsa-miR-1184	TMEM191C
hsa-miR-612	RP11-458F8.4
hsa-miR-198	RP11-830F9.6
hsa-miR-18a-3p	RP3-388N13.3
hsa-miR-1178-3p	AC011284.3
hsa-miR-2355-5p	RP11-54O7.17
hsa-miR-1224-5p	RP3-388N13.3
hsa-miR-125b-2-3p	RP11-333E1.2
hsa-miR-650	RP5-892K4.1
hsa-miR-2113	RP11-982M15.8
hsa-miR-1972	FAM182A
hsa-miR-214-3p	RP4-539M6.22
hsa-miR-939-5p	RP11-627G23.1
hsa-miR-765	RP11-627G23.1
hsa-miR-148a-3p	LA16c-306A4.2
hsa-miR-130a-3p	LA16c-306A4.2
hsa-miR-136-5p	RP11-526P6.1
hsa-miR-186-5p	MIR325HG
hsa-miR-650	AIRN
hsa-miR-7-5p	RP11-338K13.1
hsa-miR-1224-5p	KB-1183D5.13
hsa-miR-20a-3p	LINC01043
hsa-miR-1207-5p	C10orf91
hsa-miR-1224-5p	XXyac-YM21GA2.7
hsa-miR-29a-5p	MUC19
hsa-miR-1184	LINC00689
hsa-miR-1972	LINC01002
hsa-miR-1207-5p	RP11-618K13.2
hsa-miR-1972	RP11-1228E12.1
hsa-miR-18a-3p	GAS8-AS1
hsa-miR-1976	RP13-580B18.4
hsa-miR-342-3p	CTD-3138B18.5
hsa-miR-1224-5p	CTD-2619J13.14
hsa-miR-582-3p	GAS6-AS1
hsa-miR-1236-3p	RP11-717I24.1

(*Continued*)

Table S11: *Continued*

miRNA	lncRNA
hsa-miR-7-5p	RP11-932O9.4
hsa-miR-129-5p	RP11-166B2.5
hsa-miR-582-3p	LINC00969
hsa-miR-877-3p	LINC00689
hsa-miR-1972	LINC01001
hsa-miR-541-3p	AC011284.3
hsa-miR-1224-5p	AC011718.2
hsa-miR-149-5p	RP11-394A14.2
hsa-miR-30b-3p	MUC2
hsa-miR-324-5p	LINC01043
hsa-miR-342-3p	RP11-210M15.1
hsa-miR-1972	LINC00174
hsa-miR-483-3p	MUC19
hsa-miR-483-3p	CTD-2281E23.3
hsa-miR-1207-5p	LINC00265
hsa-miR-1207-5p	RP11-333E1.2
hsa-miR-612	RP11-1260E13.1
hsa-miR-198	RP11-32B5.8
hsa-miR-1184	RP3-470B24.5
hsa-miR-335-3p	SLC8A1-AS1
hsa-miR-181c-3p	RP11-627G23.1
hsa-miR-665	CTB-51J22.1
hsa-miR-939-5p	HP09025
hsa-miR-1976	AC010761.6
hsa-miR-302a-3p	RP4-539M6.22
hsa-miR-486-3p	RP11-94C24.13
hsa-miR-1207-5p	AP001476.4
hsa-miR-1207-5p	RP4-539M6.22
hsa-miR-148a-3p	RP11-717I24.1
hsa-miR-190a-5p	DPP10-AS2
hsa-miR-181a-5p	LL22NC03-27C5.1
hsa-miR-650	RP11-458F8.4
hsa-miR-18a-3p	CTD-2619J13.14
hsa-miR-223-3p	FAM95B1
hsa-miR-542-3p	RP4-671O14.7
hsa-miR-1184	HCG22
hsa-miR-1224-5p	NNT-AS1
hsa-miR-7-5p	AC006019.3
hsa-miR-541-3p	FLJ35934
hsa-miR-542-3p	AC079586.1

(*Continued*)

Table S11: *Continued*

miRNA	lncRNA
hsa-miR-561-3p	RP11-231G3.1
hsa-miR-142-3p	MUC2
hsa-miR-324-5p	RP11-54O7.17
hsa-miR-20a-3p	RP11-54O7.17
hsa-miR-1200	LINC01123
hsa-miR-1976	LINC01123
hsa-miR-27a-5p	AP000345.1
hsa-miR-939-5p	LINC00173
hsa-miR-194-3p	RP4-671O14.7
hsa-miR-18a-3p	NNT-AS1
hsa-miR-223-5p	LINC00689
hsa-miR-542-3p	RP11-157B13.7
hsa-miR-650	LINC00689
hsa-miR-1236-3p	LINC00689
hsa-miR-2113	AC005264.2
hsa-miR-129-5p	AC006548.28
hsa-miR-650	LINC00265
hsa-miR-877-3p	LINC00940
hsa-miR-214-5p	HPVC1
hsa-miR-148a-3p	RP1-182D15.2
hsa-miR-1976	LINC01523
hsa-miR-1236-3p	LINC00940
hsa-miR-223-5p	RP11-426C22.4
hsa-miR-148a-3p	SNHG14
hsa-miR-130a-3p	SNHG14
hsa-miR-1976	CTD-3032J10.2
hsa-miR-186-5p	LINC00613
hsa-miR-218-5p	RP11-526P6.1
hsa-miR-218-1-3p	LINC01043
hsa-miR-650	RP11-304L19.13
hsa-miR-18a-3p	CTD-2517M22.17
hsa-miR-223-5p	HPVC1
hsa-miR-106a-5p	LINC01106
hsa-miR-650	RP5-1014D13.2
hsa-miR-542-3p	LINC00917
hsa-miR-1184	RP11-1260E13.4
hsa-miR-1200	CTD-2008P7.3
hsa-miR-1972	CH507-216K13.2
hsa-miR-106a-5p	RP11-369C8.1
hsa-miR-539-5p	AC018816.3

(*Continued*)

Table S11: *Continued*

miRNA	lncRNA
hsa-miR-194-3p	MUC19
hsa-miR-1184	AC092535.3
hsa-miR-1207-5p	RP11-680F20.6
hsa-miR-30b-3p	LINCMD1
hsa-miR-186-5p	DYX1C1-CCPG1
hsa-miR-30b-3p	RP11-153F5.7
hsa-miR-612	AC004156.3
hsa-miR-1200	RP11-22M7.2
hsa-miR-1976	TMEM191C
hsa-miR-214-3p	TTLL10-AS1
hsa-miR-1972	CTD-2330K9.2
hsa-miR-1972	LINC00905
hsa-miR-1184	TMEM191A
hsa-miR-214-3p	AC015849.16
hsa-miR-223-5p	RP11-243A14.1
hsa-miR-1976	LINC00174
hsa-miR-1207-5p	RP11-867G23.4
hsa-miR-18a-3p	ADGRA1-AS1
hsa-miR-650	RP11-66B24.2
hsa-miR-30b-3p	AC011284.3
hsa-miR-214-3p	TMEM9B-AS1
hsa-miR-186-5p	RP11-154D6.1
hsa-miR-765	ST20-AS1
hsa-miR-650	CTD-2013N17.7
hsa-miR-194-3p	FAM95B1
hsa-miR-939-5p	VPS9D1-AS1
hsa-miR-1200	RP11-627G23.1
hsa-miR-1976	RP11-627G23.1
hsa-miR-18a-3p	RP5-1029F21.2
hsa-miR-939-5p	CTA-941F9.10
hsa-miR-665	RP13-895J2.3
hsa-miR-139-5p	AC015849.16
hsa-miR-186-5p	RP11-22A3.2
hsa-miR-939-5p	RP11-311F12.1
hsa-miR-590-3p	LINC00240
hsa-miR-186-5p	SFTPD-AS1
hsa-miR-18a-3p	RP11-573D15.2
hsa-miR-18a-3p	RP11-469N6.1
hsa-miR-1207-5p	LINC00969
hsa-miR-650	RP5-1039K5.19

(*Continued*)

Table S11: *Continued*

miRNA	lncRNA
hsa-miR-1976	LINC01054
hsa-miR-18a-3p	AP001062.7
hsa-miR-612	LINC00664
hsa-miR-665	RP13-582L3.4
hsa-miR-1972	RP11-142C4.6
hsa-miR-129-5p	LINC00662
hsa-miR-182-5p	RP11-34P13.7
hsa-miR-1207-5p	H19
hsa-miR-1207-5p	RP5-1142A6.2
hsa-miR-7-5p	RP11-394A14.2
hsa-miR-1200	RP11-1129I3.1
hsa-miR-205-5p	FAR1-IT1
hsa-miR-542-3p	LINC01224
hsa-miR-612	MIRLET7BHG
hsa-miR-939-5p	RP11-278A23.4
hsa-miR-335-3p	CTA-392E5.1
hsa-miR-590-3p	AC005614.3
hsa-miR-190a-5p	LINC00664
hsa-miR-181c-3p	SNHG14
hsa-miR-223-3p	RP1-182D15.2
hsa-miR-20a-3p	LINC00906
hsa-miR-539-5p	ZNF883
hsa-miR-198	CTA-390C10.9
hsa-miR-539-5p	CTC-435M10.10
hsa-miR-136-5p	SPACA6P
hsa-miR-1972	RP11-504P24.8
hsa-miR-18a-3p	CTD-2245F17.9
hsa-miR-1184	CTD-3193O13.11
hsa-miR-1976	CTB-60B18.18
hsa-miR-129-5p	RP11-69I8.2
hsa-miR-1972	RP11-849H4.4
hsa-miR-1236-3p	RP11-798K23.1
hsa-miR-218-1-3p	RP11-211G23.2
hsa-miR-139-5p	RP11-231D20.2
hsa-miR-182-5p	FENDRR
hsa-miR-513a-3p	RP11-474P2.6
hsa-miR-30b-3p	TTLL10-AS1
hsa-miR-665	SNHG14
hsa-miR-650	RP11-378E13.3
hsa-miR-765	AC005324.6

(*Continued*)

Table S11: *Continued*

miRNA	lncRNA
hsa-miR-939-5p	RP5-1171I10.5
hsa-miR-1224-5p	CTD-2245F17.9
hsa-miR-1224-5p	LINC00689
hsa-miR-1200	LINC00689
hsa-miR-650	CTD-2283N19.1
hsa-miR-1976	CTD-2135D7.2
hsa-miR-335-3p	RP11-146D12.2
hsa-miR-665	CH17-360D5.1
hsa-miR-182-5p	AC010524.2
hsa-miR-335-3p	RP11-335L23.4
hsa-miR-181c-3p	CTD-2281E23.3
hsa-miR-1236-3p	RP11-91K11.2
hsa-miR-7-5p	LINC00662
hsa-miR-2355-5p	RP11-161M6.2
hsa-miR-30b-3p	RP11-94C24.13
hsa-miR-1184	RP1-29C18.10
hsa-miR-921	RP11-326C3.14
hsa-miR-1236-3p	RP11-23J9.4
hsa-miR-590-3p	RP11-762H8.4
hsa-miR-186-5p	RP1-288H2.2
hsa-miR-1184	CTD-2311B13.1
hsa-miR-27a-5p	AC004156.3
hsa-miR-342-3p	RP13-580B18.4
hsa-miR-485-5p	AP001626.2
hsa-miR-214-3p	CTC-242N15.1
hsa-miR-130a-3p	RP4-539M6.22
hsa-miR-541-3p	CTD-2619J13.14
hsa-miR-650	AC074212.5
hsa-miR-939-5p	AP001469.9
hsa-miR-612	LINC01015
hsa-miR-650	CTA-243E7.4
hsa-miR-129-5p	RP11-67K19.3
hsa-miR-1976	CTD-2126E3.3
hsa-miR-765	ATP2A1-AS1
hsa-miR-186-5p	AC124997.1
hsa-miR-149-5p	CTD-2008P7.3
hsa-miR-1207-5p	LINC01168
hsa-miR-541-3p	AP001626.2
hsa-miR-129-5p	REV3L-IT1
hsa-miR-939-5p	RP11-186N15.3

(*Continued*)

Table S11: *Continued*

miRNA	lncRNA
hsa-miR-612	AJ011931.1
hsa-miR-665	AC114808.3
hsa-miR-1976	RP11-203B9.4
hsa-miR-590-3p	AC006548.28
hsa-miR-1972	RP11-1191J2.2
hsa-miR-30b-3p	RP11-244B22.11
hsa-miR-612	RP11-413M3.4
hsa-miR-335-3p	LINC01122
hsa-miR-590-3p	AC093639.1
hsa-miR-1207-5p	AC000095.11
hsa-miR-485-5p	LINC00265
hsa-miR-214-3p	NR2F1-AS1
hsa-miR-650	EIF3J-AS1
hsa-miR-129-5p	RP5-1125A11.7
hsa-miR-660-5p	RP1-29C18.9
hsa-miR-1184	AC139099.4
hsa-miR-214-5p	RP11-384K6.6
hsa-miR-186-5p	CTD-2410N18.4
hsa-miR-214-3p	CTC-321K16.1
hsa-miR-539-5p	SATB1-AS1
hsa-miR-129-5p	RP3-508I15.22
hsa-miR-541-3p	IDI2-AS1
hsa-miR-18a-3p	UCKL1-AS1
hsa-miR-335-3p	RP11-96K19.4
hsa-miR-877-3p	RP1-253P7.1
hsa-miR-186-5p	AJ003147.8
hsa-miR-539-5p	LINC01539
hsa-miR-1972	LINC00661
hsa-miR-1184	COL18A1-AS1
hsa-miR-2355-5p	LA16c-306A4.2
hsa-miR-18a-3p	CTD-2523D13.1
hsa-miR-509-3p	CTD-2278I10.1
hsa-miR-129-5p	SEPSECS-AS1
hsa-miR-9-5p	RP11-397O4.1
hsa-miR-186-5p	CTD-3046C4.1
hsa-miR-650	RP3-395M20.8
hsa-miR-186-5p	RP11-227H15.4
hsa-miR-1184	RP3-402G11.28
hsa-miR-1976	RP11-304L19.3
hsa-miR-944	RP5-1077H22.2

(*Continued*)

Table S11: *Continued*

miRNA	lncRNA
hsa-miR-513a-3p	LL22NC03-N64E9.1
hsa-miR-1976	LINC01001
hsa-miR-612	FLJ26245
hsa-miR-612	AC079586.1
hsa-miR-541-3p	RP4-545C24.1
hsa-miR-539-5p	RP11-598F7.3
hsa-miR-541-3p	AP006621.9
hsa-miR-938	RP11-989E6.10
hsa-miR-612	SHANK3
hsa-miR-612	AC092171.4
hsa-miR-129-5p	RP11-848P1.3
hsa-miR-186-5p	LINC00662
hsa-miR-590-3p	CTD-2561J22.5
hsa-miR-129-5p	RP11-486O12.2
hsa-miR-30b-3p	RP11-480I12.10
hsa-miR-186-5p	CTB-181F24.1
hsa-miR-665	RASSF8-AS1
hsa-miR-485-5p	RP11-384K6.6
hsa-miR-18a-3p	LL22NC03-86G7.1
hsa-miR-129-5p	RP1-283E3.8
hsa-miR-708-3p	LINC00662
hsa-miR-1202	SNHG14
hsa-miR-7-5p	CTA-243E7.1
hsa-miR-129-5p	RP11-189E14.3
hsa-miR-486-3p	RP4-539M6.22
hsa-miR-513a-3p	AC009299.3
hsa-miR-485-5p	AC138035.2
hsa-miR-214-5p	RP13-580B18.4

(*Continued*)

Table S11: *Continued*

miRNA	lncRNA
hsa-miR-590-3p	LA16c-60D12.2
hsa-miR-181a-5p	ZNF833P
hsa-miR-1972	FAM95B1
hsa-miR-342-3p	AC078942.1
hsa-miR-939-5p	LINC00599
hsa-miR-539-5p	EGFLAM-AS3
hsa-miR-650	LA16c-313D11.12
hsa-miR-1207-5p	MIRLET7BHG
hsa-miR-377-5p	AP000442.1
hsa-miR-1976	RP11-33B1.4
hsa-miR-186-3p	RP11-368I7.4
hsa-miR-765	RP11-570L14.2
hsa-miR-20a-3p	CTD-2066L21.2
hsa-miR-214-3p	LINC01304
hsa-miR-650	RP5-1171I10.5
hsa-miR-129-5p	RP4-794I6.4
hsa-miR-186-5p	SNHG14
hsa-miR-590-3p	RP11-638L3.1
hsa-miR-922	LA16c-306A4.2
hsa-miR-1205	RP3-470B24.5
hsa-miR-342-3p	LINC01002
hsa-miR-18a-3p	RP11-54O7.17
hsa-let-7f-2-3p	FAM230B
hsa-miR-922	RP4-539M6.22
hsa-miR-92a-2-5p	CTD-2619J13.14
hsa-miR-1976	RP11-248M19.1
hsa-miR-214-5p	RP11-804H8.7
hsa-miR-654-5p	PAX8-AS1
